# A new, large-bodied omnivorous bat (Noctilionoidea: Mystacinidae) reveals lost morphological and ecological diversity since the Miocene in New Zealand

**DOI:** 10.1038/s41598-017-18403-w

**Published:** 2018-01-10

**Authors:** Suzanne J. Hand, Robin M. D. Beck, Michael Archer, Nancy B. Simmons, Gregg F. Gunnell, R. Paul Scofield, Alan J. D. Tennyson, Vanesa L. De Pietri, Steven W. Salisbury, Trevor H. Worthy

**Affiliations:** 1 PANGEA Research Centre, School of Biological, Environmental and Earth Sciences, UNSW Australia, Sydney, 2052 Australia; 2 School of Environment and Life Sciences, University of Salford, Salford, M5 4WT UK; 3 Department of Mammalogy, Division of Vertebrate Zoology, American Museum of Natural History, New York, New York, 10024 USA; 4 Division of Fossil Primates, Duke University Lemur Center, Durham, North Carolina 27705 USA; 5 Canterbury Museum, Rolleston Ave, Christchurch, 8001 New Zealand; 6 Museum of New Zealand Te Papa Tongarewa, Wellington, 6140 New Zealand; 7 School of Biological Sciences, The University of Queensland, Brisbane, 4072 Australia; 8 Biological Sciences, College of Science and Engineering, Flinders University, Adelaide, 5001 Australia

## Abstract

A new genus and species of fossil bat is described from New Zealand’s only pre-Pleistocene Cenozoic terrestrial fauna, the early Miocene St Bathans Fauna of Central Otago, South Island. Bayesian total evidence phylogenetic analysis places this new Southern Hemisphere taxon among the burrowing bats (mystacinids) of New Zealand and Australia, although its lower dentition also resembles Africa’s endemic sucker-footed bats (myzopodids). As the first new bat genus to be added to New Zealand’s fauna in more than 150 years, it provides new insight into the original diversity of chiropterans in Australasia. It also underscores the significant decline in morphological diversity that has taken place in the highly distinctive, semi-terrestrial bat family Mystacinidae since the Miocene. This bat was relatively large, with an estimated body mass of ~40 g, and its dentition suggests it had an omnivorous diet. Its striking dental autapomorphies, including development of a large hypocone, signal a shift of diet compared with other mystacinids, and may provide evidence of an adaptive radiation in feeding strategy in this group of noctilionoid bats.

## Introduction

The main islands of New Zealand are the largest emergent part of the continental fragment of Zealandia, other landmasses of which today include New Caledonia, Lord Howe, Chatham and Campbell Islands^1,2^. Zealandia separated from the Australia-Antarctica part of Gondwana in a split that began 130 Ma (million years ago), with the Tasman Sea opening from south to north in the interval 83–52 Ma^3,4^ and with ~1600 km of ocean now separating Australia and New Zealand. Australia, Antarctica and South America remained connected until ~40 Ma, as the last vestiges of Gondwana^5–7^.

Today, New Zealand has a biogeographically highly distinctive fauna that includes many old endemic lineages and recent immigrants, with both vicariance and dispersal implicated in its assembly^8,9^. Its modern terrestrial mammal fauna comprises three bat species, all other modern mammals having been introduced during the last 800 years^10^. *Chalinolobus tuberculatus*, of the cosmopolitan bat family Vespertilionoidae, is closely related to its Australian congeners and probably made a trans-Tasman crossing from Australia less than 2 Ma^11^. The other two Recent bat species, *Mystacina tuberculata* and *M. robusta*, are the only living members of the family Mystacinidae. These are morphologically and ecologically very distinctive chiropterans, also known as burrowing bats, which spend 30% of their foraging time on the forest floor, under leaf litter and on tree branches^12^. *Mystacina tuberculata* is considered vulnerable to extinction and *M. robusta* critically endangered or extinct^13,14^.

Mystacinidae is one of the six to seven extant families that make up the bat superfamily Noctilionoidea, along with the Neotropical families Phyllostomidae, Noctilionidae, Mormoopidae, Furipteridae and Thyropteridae^15^. Madagascar’s Myzopodidae is also typically included in Noctilionoidea as sister to the remaining families (e.g.^16,17^), but some analyses of molecular data suggest it has a sister-group relationship with Vespertilionoidea (e.g.^18,19^), or that it is sister to Emballonuroidea, or (within Emballonuroidea) Nycteridae^20^.

With or without Myzopodidae included, Noctilionoidea is the only bat superfamily interpreted to have a Gondwanan origin^16^. The noctilionoid fossil record is poor, especially for the Paleogene^21,22^, but biogeographic reconstructions suggest that this morphologically and ecologically diverse superfamily probably originated in Africa (e.g.^18,22,23^), with subsequent dispersal and radiation producing Australasia’s mystacinids and the five modern Neotropical noctilionoid families. According to molecular data, the divergence of the Australasian and South American noctilionoid clades occurred ~50–37 Ma^20,24,25^.

Fossils show that mystacinids once occurred in Australia (26–12 Ma; refs^26,27^) and were present in New Zealand from at least the early Miocene^28^. In New Zealand, remains of the two modern *Mystacina* species have been recovered from numerous Pleistocene and Holocene cave deposits^29^. The Miocene mystacinid *Mystacina miocenalis* has been described^30^ and material indicative of two smaller mystacinid species has been reported^28^ from freshwater lake sediments (16–19 Ma) near St Bathans, Central Otago, South Island. The St Bathans fossil assemblage also includes plants, invertebrates, fish, frogs, lizards, kiwi, moa, New Zealand wrens, parrots, waders and many other water birds, a tuatara, crocodilian and turtle, and fragments of a small non-volant archaic mammal (e.g.^9,31–37^). As Zealandia’s only known Tertiary terrestrial vertebrate fauna, the St Bathans fossil assemblage offers critical insight into the deep-time history for most of its vertebrate lineages.

Here, we describe a new bat genus and species from St Bathans, and discuss its bearing on hypotheses regarding the radiation of the southern superfamily Noctilionoidea and the family Mystacinidae in the Australian region. This fossil bat indicates that there once was greater ecological diversity in the New Zealand’s bat fauna, and, as only the third bat genus recorded from New Zealand, it signals substantial loss of diversity since the Miocene.

## Systematic palaeontology

Order Chiroptera Blumenbach, 1779

Suborder **Yangochiroptera** Van den Bussche & Hoofer, 2004

Superfamily **Noctilionoidea** Gray, 1821

Family **Mystacinidae** Dobson, 1875

***Vulcanops jennyworthyae*** gen. et sp. nov.

(Figs 1–2)

**Figure 1:**
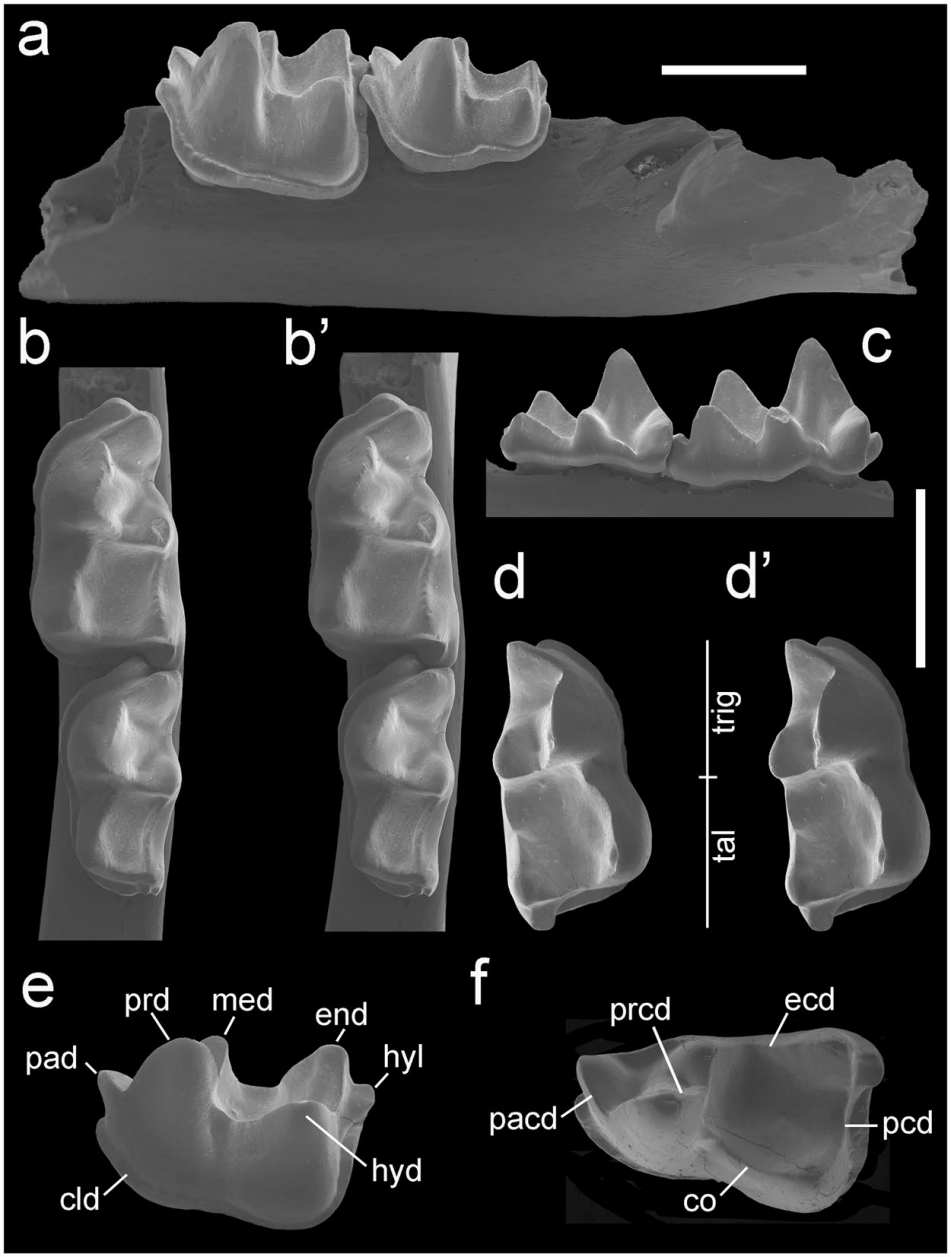
*Vulcanops jennyworthyae* gen. et sp. nov., Bannockburn Formation, St Bathans, Central Otago, New Zealand. Lower dentition. CM 2013.18.790, holotype, left dentary fragment containing m2-3. (**a**) Buccal view; (**b–b**’) stereopair, occlusal view; (**c**) lingual view m2-3. NMNZ S.52078, paratype, right m1. (**d–d**’) Stereopair, oblique occlusal view; (**e**) buccal view; (**f**) occlusal view. Abbreviations: cld, cingulid; co, cristid obliqua; end, entoconid; ecd, entocristid; hyd, hypoconid; hyl, hypoconulid; med, metaconid; pacd, paracristid; pad, paraconid; pcd, postcristid; prcd, protocristid; prd, protoconid; tal, talonid; trig, trigonid. Scale bars = 2 mm.

**Figure 2:**
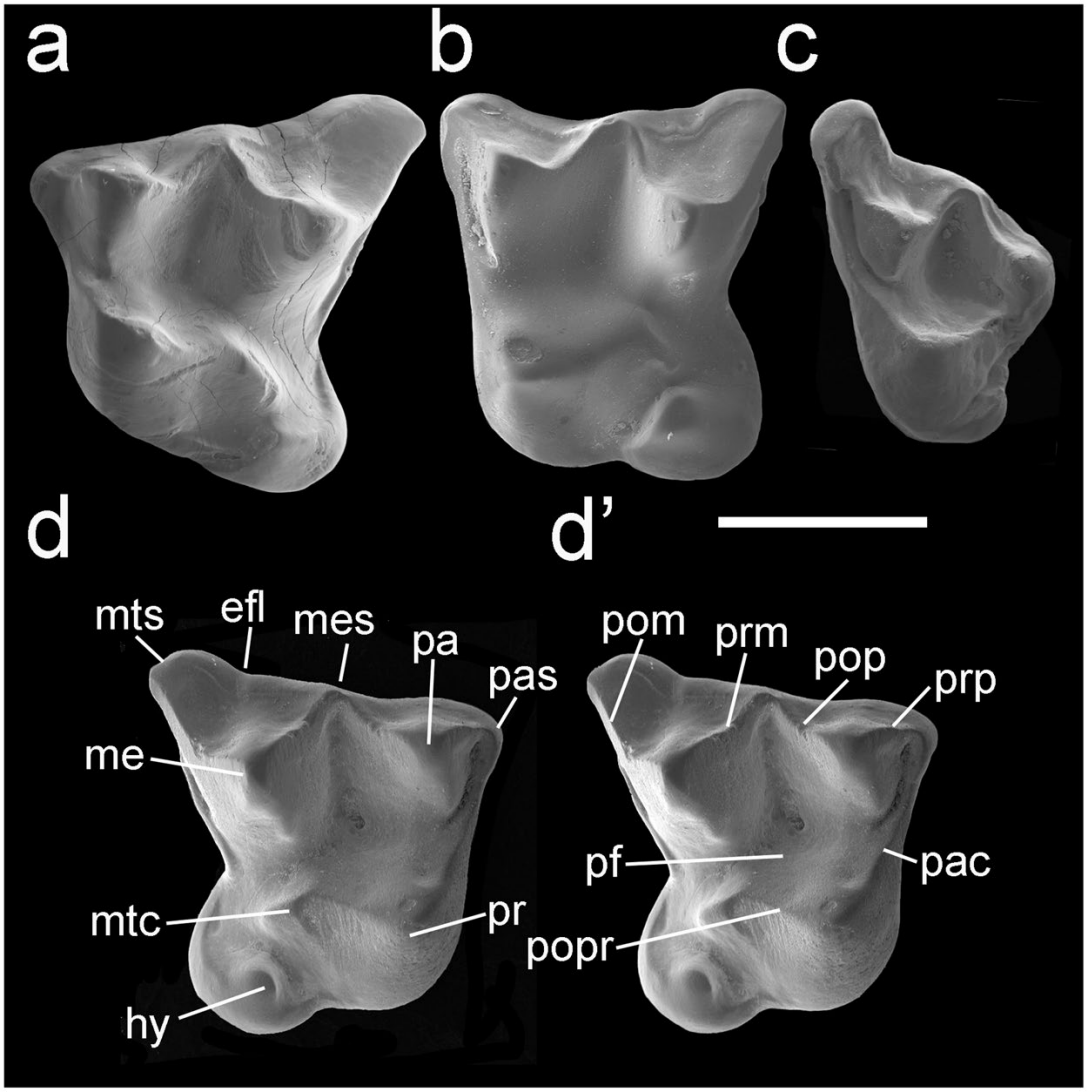
*Vulcanops jennyworthyae* gen. et sp. nov. Upper dentition. (**a**) CM 2013.18.916, left M1, oblique occlusal view. (**b**) NMNZ S.50383, right M2, oblique occlusal view (reversed). (**c**) NMNZ S.52400, left M3, oblique occlusal view. (**d–d**’) NMNZ S.44071, right M1, stereo-pair, oblique occlusal view. Abbreviations: efl, ectoflexus; hy, hypocone; me, metacone; mes, mesostyle; mtc, metaconule; mts, metastyle; pa, paracone; pac, paracingulum; pas, parastyle; pf, protofossa; pr, protocone; prp preparacrista; prm, premetacrista; pom, postmetacrista; pop, postparacrista; popr, postprotocrista. Scale bar = 2 mm.

### Generic diagnosis

As for the type and only species.

### Stratigraphic and geographic distribution

Lower Miocene of Central Otago, New Zealand.

### Etymology

From *Vulcan*, mythological god of fire and volcanoes (Roman), and *ops*, a suffix commonly used for bats; in reference to New Zealand’s tectonically active nature, as well as to the historic Vulcan Hotel, centre of the hamlet of St Bathans, from which the fauna takes its name. The species name honours Jennifer P. Worthy in recognition of her pivotal role in revealing the diversity of the St Bathans Fauna.

### Holotype

CM 2013.18.790, left dentary fragment with m2-3 (Fig. 1a–c), HH1a, Bannockburn Formation, Manuherikia River, Home Hills Station, Otago, New Zealand (see *Locality and age*).

### Referred specimens

NMNZ S.42876, right m1, HH1a; NMNZ S.52078, right m1 (Fig. 1d–f), HH1a; NMNZ S.52076, left m1/2 (fragment), HH1a; CM 2013.18.916, left M1, HH1a (Fig. 2a); NMNZ S.44071, right M1, HH1a (Fig. 2d); NMNZ S.51461, right M1, HH1b Trench; CM 2013.18.1, left M1, Croc Site Layer 1; NMNZ S.51746, left M2, HH1b Trench; NMNZ S.50383, right M2, HH1a (Fig. 2b); NMNZ S.50778, right M1/2 (posterolingual fragment), HH1a; NMNZ S.52400, left M3, HH1b Trench (Fig. 2c); NMNZ S.52351, right incomplete M3, HH1a; NMNZ S.50384, left M3 incomplete, HH1a. A minimum of four individuals is represented. Measurements of the fossils are given in Table 1.

**Table 1:** Measurements (mm) of lower molars (m) and upper molars (m) of *Vulcanops jennyworthyae* gen. et sp. nov. from the lower Miocene Bannockburn Formation, St Bathans, Central Otago, New Zealand. *Figured, ^‡^holotype.

Specimen no.	Position	Length	Width	Trig. width	Tal. width
NMNZ S.52078	m1	2.90		1.55	1.70
NMNZ S.42876*	m1	2.90		1.45	1.80
CM 2013.18.790*^‡^	m2-3	5.30			
	m2	2.80		1.80	2.00
	m3	2.55		1.55	1.30
NMNZ S.51461	M1	2.95	3.00		
NMNZ S.44071*	M1	2.85	2.85		
CM2013.18.1	M1	[3.00]			
CM 2013.18.916*	M1	3.05	3.10		
NMNZ S.50383*	M2	2.95	3.15		
NMNZ S.52400*	M3	2.25	2.80		

### Locality and age

Bed HH1a (New Zealand Fossil Record File number H41/f088), a 5–10 cm thick sandy conglomerate, 6.88–7.0 m above base of Bannockburn Formation, Manuherikia River section, Home Hills Station, St Bathans, Otago, New Zealand; 44.907944°S, 169.858222°E. HH1b Trench (H41/f0103), a 10 cm thick sandy conglomerate, 9.5–9.58 m above the base of the Bannockburn Formation, foot of hill 50 m across terrace from river bank, Manuherikia River section, Home Hills Station, Otago; 44.90780°S; 169.85844°E. Croc Site, Layer 1 (H41/f084), c.10 cm thick sand and cobble layer, in 3 m cliff on the north slope of a small hill on the west side of Mata Creek, Dunstanburn Station, St Bathans, Otago; 44.889500°S 169.837833°E. Altonian local stage, lower Miocene, 19–16 Ma^35^.

### Species diagnosis

A bat with: m1-2 myotodont, with talonid longer and conspicuously wider than trigonid (particularly on m1), and rounded talonid basin; m1-2 paraconid buccally displaced, not aligned with metaconid and entoconid; m1-2 entoconid very tall, with pre-entocristid interrupted such that talonid opens lingually; m1-3 cristid obliqua curved, with inflection close to trigonid, contacting trigonid conspicuously buccal to midpoint between protoconid and metaconid; m1-3 with complete anterior, buccal and posterior cingulid; m1-3 with relatively shallow hypoflexid; m3 reduced in length and width, talonid narrower than trigonid, myotodont with small hypoconulid; M1-2 as wide as long, three rooted with anteroposteriorly extended lingual root, paracone reduced in volume but subequal in height with protocone, metacone taller, parastyle conical, non-cuspidate mesostyle on buccal margin of crown, postmetacrista elongated, large posterolingually directed heel (hypocone shelf) bearing tall, bulbous hypocone, metaconule in postprotocrista with short posterolingual crest not reaching hypocone, protofossa long, deep and open posteriorly, narrow paracingulum present, posterior cingulum indistinct, lacking paraloph, metaloph and anterolingual cingulum; M3 large, metacone with complete premetacrista but no postmetacrista, heel (hypocone shelf) with small hypocone. An expanded description is given in the Supplementary Information online.

### Differential diagnosis

Differs from other mystacinids (species of *Mystacina* and *Icarops*) in exhibiting the following traits: m1-2 paraconid buccally displaced, not aligned with metaconid and entoconid, with talonid conspicuously wider than trigonid; m1-3 cristid obliqua curved rather than straight, with inflection near trigonid, contacting trigonid buccal rather than at midpoint between protoconid and metaconid; m1-3 with only shallow hypoflexid; m3 more reduced in length and width; M1-2 with hypocone present and conical parastyle; M3 long with broad angles between ectoloph cristae and with hypocone shelf and hypocone present. Differs additionally from *Mystacina* spp. in M1-2 having long, wide heel (hypocone shelf).

Differs from myzopodids in having: m1-3 cristid obliqua curving lingually rather than buccally; all trigonids with equally wide trigonid angle; m3 with small hypoconulid present. Differs additionally from *Myzopoda* spp. in having: M1-3 with hypocone shelf and hypocone; M1-2 as wide as long, with broader angles between ectoloph cristae, preparacrista shorter than postparacrista and postmetacrista elongated, ectocingulum variably present but indistinct.

Differs from thyropterids and furipterids in having: m1-2 paraconid buccally displaced, not aligned with metaconid and entoconid; cristid obliqua contacting trigonid conspicuously buccal to midpoint between protoconid and metaconid; m1-3 with only shallow hypoflexid; m3 reduced in length and width; M1-3 lacking paraloph and metaloph; M1-2 with hypocone shelf and hypocone; M1-2 long with broader angles between ectoloph cristae; postmetacrista elongated; presence of ectocingulum; lacking buccally extruded mesostyles (deep ectoflexi?); M1-2 without continuous lingual cingulum. Additionally differs from furipterids in its M1-2 lacking metacingulum; m1-3 myotodont rather than nyctalodont, without conical entoconid and lacking postmetacristid. Additionally differs from thyropterids in its M3 smaller (e.g. narrower) than M1.

Differs from noctilionids in its: m1-3 without tall, long/continuous entocristid closing talonid; with only shallow hypoflexid; m1-3 trigonids not anteroposteriorly compressed, with protocristid and cristid obliqua curved, the latter lingually with inflection near trigonid, contacting trigonid conspicuously buccal to midpoint between protoconid and metaconid (rather than extending to lingual margin of crown); M1-2 with postparacrista and premetacrista meeting on buccal margin such that centrocrista continuous; M1-2 with rounded (rather than sharply/pointed) posterolingually directed and unbasined heel; lacking strong paraloph and metaloph that close the protofossa anteriorly and posteriorly; M3 with hypocone shelf and small hypocone, lacking paraloph.

Differs from mormoopids in its: m1-3 cristid obliqua meeting trigonid buccal of centre; talonid basin rounded rather than triangular (with lingually curved rather than straight cristid oblique); M1-2 with shorter, posterolingually (rather than posteriorly) directed heel; angles between ectostyle cristae wider; without hooked parastyle; incomplete/absent lingual cingulum; M3 with hypocone.

Differs from desmodontine, stenodermatine, carolliine and rhinophylline phyllostomids in having dilambdodont molars. Differs from most phyllostomines, macrotines, micronycterines, lonchophyllines, lonchorhynines and glossophagines in its myotodont rather than nyctalodont lower molars. Differs additionally from phyllostomines in M3 being large and with hypocone.

### Body mass

Using the equations of Gunnell *et al*.^38^ and the proxies of upper first molar (M1) area, lower first molar (m1) area, and diameter of mid-shaft humerus, the body mass of eight of the ten known extinct and extant mystacinid taxa are given in Table 2. For the previously known mystacinids, these values range from ~8.5 g (*Icarops paradox*) to 39.3 g (*Mystacina miocenalis*). For *Vulcanops jennyworthyae*, the estimates are 42.6 g (based on M1 area) and 39.8 g (m1 area). This indicates a relatively large bat, compared with the median value of 13.8 g for 905 extant bat species (refs^38,39^; see Discussion).

**Table 2:** Body mass estimates (g) of extinct and extant mystacinids from New Zealand (NZ) and Australia (Aus) based on equations in Gunnell *et al*.^22^ and using the proxies of upper first molar (M1) area, lower first molar (m1) area, and humerus mid-shaft diameter.

Taxon	Location	Age	Body mass estimate			Live weight
			M1	m1	humerus	
†*Vulcanops jennyworthyae* ^1^	NZ	E Miocene	42.6 (1)	39.8 (1)	—	—
†*Mystacina miocenalis* ^2^	NZ	E Miocene	39.3 (1)	—	—	—
†Mystacinid indet. 1^3^	NZ	E Miocene	—	—	114.33 (1)	—
*Mystacina tuberculata* ^4^	NZ	Holocene	12.77 (20)	14.28 (20)	112.23 (5)	13.6 (300)
*Mystacina robusta* ^5^	NZ	Holocene	22.90 (12)	22.19 (12)	117.70 (1)	—
†*Icarops paradox* ^6^	Aus	E Miocene	8.43 (2)	10.31 (1)	—	—
†*Icarops aenae* ^7^	Aus	L Oligo-E Mio	17.74 (2)	21.52 (1)	115.07 (2)	—
†Mystacinid indet.^8^	Aus	L Oligocene	11.34 (1)	—	—	—

† Indicates extinct taxon; E, Early; L, Late; (#), number of specimens. Humerus mid-shaft measured in this work. Dental and weight data from: **1**, this paper (Table 1); **2**,^30^; **3**,^28^; **4**,^87^ (Codfish Is),^73^; **5**, ^30,87^ (Stewart Is); **6 & 7**,^27^; **8**,^45^. No estimates available for *Icarops breviceps* (known from m2-3;^26^) but tooth size similar to *I. aenae* ^27^, nor Mystacinid indet. 2 but distal humerus is smaller than in Mystacinid indet. 1^28^.

## Phylogeny

**Figure 3:**
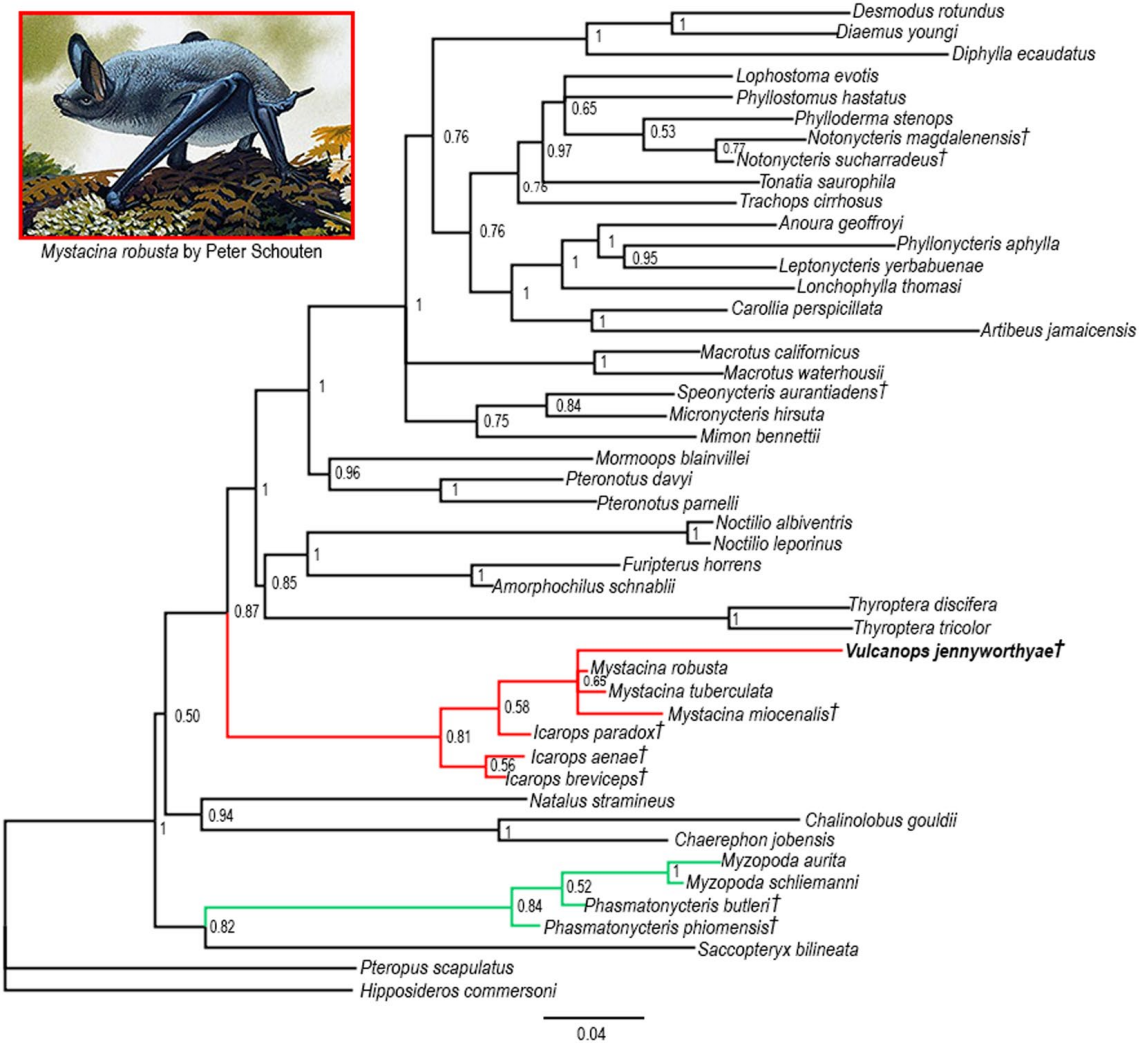
50% majority rule consensus tree of post-burn-in trees from Bayesian total evidence analysis of 292 dental characters plus 11.1 kb of mitochondrial and nuclear DNA sequence data. Values at nodes represent Bayesian posterior probabilities values > 0.5. † Indicates extinct taxon; green, Myzopodidae; red, Mystacinidae + *Vulcanops*. Illustration of *Mystacina robusta* by Peter Schouten.

The 50% majority rule consensus of post-burn-in trees from our Bayesian total evidence analysis is given in Fig. 3. Mystacinidae, Furipteridae + Noctilionidae, Thyropteridae, and Mormoopidae + Phyllostomidae formed clades, all with relatively high support (posterior probabilities shown in Fig. 3). Yangochiroptera had 100% support; Noctilionoidea and Vespertilionoidea were sister groups but with low support (50%). Myzopodidae and the emballonurid *Saccopteryx bilineata* grouped with relatively high support of 82%. *Vulcanops* fell within Mystacinidae, with a relatively high posterior probability of 81% but with relationships within the family less strongly supported (posterior probabilities 55–65%). Of the fossil taxa, *Speonycteris aurantiadens* grouped with phyllostomids rather than mormoopids^40,41^, but the others grouped in agreement with the results of previous studies, namely *Phasmatonycteris* spp. with *Myzopoda* spp. in Myzopodidae^22^, Australian *Icarops* spp. with New Zealand *Mystacina* spp.^27,30^ and *Notonycteris* spp. with phyllostomine phyllostomids^42,43^.

The Bayesian analysis identified eight unequivocal synapomorphies uniting Mystacinidae (i.e. *Icarops* + *Mystacina* + *Vulcanops*), of which four can be scored in *Vulcanops*: ectocingulum present but weak (character 71:1), secondary cusp present on postprotocrista of M1 and M2 (character 92:1), m1 hypoflexid shallow (character 252:1), and m3 cristid obliqua contacts trigonid (character 271:0). A full list of synapomorphies for all nodes in the topologies shown in Fig. 3, under both Accelerated Transformation (ACCTRAN) and Delayed Transformation (DELTRAN), is given in Supplementary Information.

## Discussion

Bayesian total evidence analysis (mitochondrial and nuclear genes plus dental characters) places the New Zealand Miocene bat *Vulcanops jennyworthyae* among Australasia’s living and fossil mystacinids. The overall results of our phylogenetic analysis are broadly congruent with recent large-scale molecular studies of bats^17,19,20,24,25^. Like some of these studies (e.g.^20^), our analysis raises questions about the inclusion of Africa’s Myzopodidae within Southern Hemisphere Noctilionoidea, suggesting instead that myzopodids may be more closely related to cosmopolitan emballonurids.

Our analysis finds a sister-group relationship between Madagascar’s extant *Myzopoda* species and North Africa’s *Phasmatonycteris* species, supporting referral of those fossil taxa to the family Myzopodidae^22^. These fossil taxa were described by Gunnell *et al*.^22^ from the Eocene Birket Qarun (~37 Ma) and Oligocene Upper Jebel Qatrani (~30 Ma) Formations of the Fayum in Egypt and referred to Myzopodidae on the basis of their lower dentitions (upper teeth are unknown). Although there are similarities between *Vulcanops* and myzopodids in the morphology of the lower dentition (e.g. m1-2 paraconid buccally displaced, not aligned with metaconid and entoconid, with talonid conspicuously wider than trigonid; m1-3 cristid obliqua curved rather than straight, with inflection near trigonid, and contacting trigonid buccal to rather than at midpoint between protoconid and metaconid; m1-3 with only shallow hypoflexid; m3 reduced in length and width with respect to m1-2; see Differential diagnosis), our phylogenetic analysis indicates these similarities are likely homoplastic.

Unequivocal noctilionoid families, from the Americas and Australasia, first appear in the fossil record slightly later: mormoopids 32–30 Ma in Florida^44^, mystacinids 26 Ma in South Australia^27,45^, phyllostomids 21 Ma in Panama^46^, and noctilionids and thyropterids 13–12 Ma in Colombia^47,48^. Furipterids are first recorded from the Pleistocene of Brazil, French Guiana, and Peru^49^. Older dates for the divergence of these lineages are estimated from recent molecular clock analyses (which use fossils as calibrations): Mystacinidae at 50.3 to 37.3 Ma^20,24,25^, and the base of the neotropical noctilionoid radiation (Thyropteridae + Furipteridae + Noctilionidae + Mormopteridae + Phyllostomidae) at 47.0 to 37.3 Ma^17,25^.

With respect to Southern Hemisphere palaeogeography, these divergence times long postdate estimated dates for the separation of India-Madagascar and Africa from Gondwana (>100 Ma), with Madagascar isolated in the Indian Ocean for more than 80 Ma^50^. The divergence dates, however, span those estimated for the breakup of the Australia-Antarctica-South America landmass, with Australia and Antarctica separating ~45 Ma and South America and Antarctica ~41 Ma^5,6^. New Zealand has been isolated in the South Pacific from ~52 Ma^3,4^, possibly before the divergence of the mystacinid lineage from other noctilionoids.

Based on phylogenetic inference and tectonic events, a number of biogeographic hypotheses have been proposed to explain the modern distribution of noctilionoids in the Southern Hemisphere. These include: a trans-Atlantic dispersal of stem noctilionoids from Africa to North or South America in the Eocene (e.g.^18,23^); a North American origin (or transit) of stem noctilionoids, with dispersal to South America via an Eocene proto-Caribbean archipelago (e.g.^21^); or an American origin or transit with subsequent dispersal of ancestral mystacinids to Australasia (e.g.^51^). Gunnell *et al*.^22^ proposed that noctilionoids originated and initially diversified in Africa (giving rise there to myzopodids) with a subsequent dispersal to Australia (producing mystacinids) and then to South America via Antarctica (this lineage leading to the five neotropical noctilionoid families).

Even if myzopodids are not noctilionoids, as suggested by some recent molecular data and by our total evidence analysis, one of these scenarios may still be valid. The modern bat crown-clade is thought to have originated in either Africa^52–54^ or Eurasia^55^, with estimates for the age of the base of the extant bat radiation ranging from 62.6 Ma^20^ to 50.3 Ma^25^. Potential living sister-groups of Noctilionoidea (*sensu* 20, i.e. excluding Myzopodidae) are vespertilionoids and emballonuroids. These two speciose groups have cosmopolitan distributions, occurring on all continents except Antarctica today, but molecular data suggest their roots were in Africa (stem and crown) and their oldest fossils are from North Africa^54^. These data, and an estimated divergence time of ~50 Ma to 37 Ma for Noctilionoidea^20,24,25^, are not inconsistent with the many previous biogeographical hypotheses for the distribution of superfamily Noctilionoidea outlined above.

The data are also potentially consistent with a vicariant origin of Mystacinidae (e.g.^56^). In the early Paleogene, global temperatures were up to 12 °C higher than today, mainland Antarctica supported a frost-free, paratropical flora until 50 Ma and *Nothofagus* forests until at least 15 Ma, and intercontinental distances in the Southern Hemisphere were generally less than now^57,58^. The Paleogene remnants of Gondwana may have supported a broadly distributed noctilionoid fauna. If so, final fragmentation of the supercontinent may have led to the extinction of noctilionoids in Neogene Antarctica as ice-sheets grew^59^, with Mystacinidae vicariantly isolated in the Australian region. However, fossil bats have yet to be found in Antarctica, and a divergence date for mystacinids from other noctilionoids of ~50 to 37 Ma, after isolation of New Zealand in the Pacific ~52 Ma^3,4^, suggests that their presence in at least New Zealand probably reflects one or more post-Gondwanan dispersals.

Other bats were present in the Australian region in the early Paleogene, as demonstrated by the archaic *Australonycteris clarkae* from the 55 Ma Tingamarra fauna of southeastern Queensland, Australia^60,61^. The likely route taken by the first bats to reach Australia is unknown (the relationship of *Australonycteris* to other early chiropterans from Northern and Southern Hemispheres is unclear^62^;). Between 55 and 26 Ma, there is long gap in the Australian mammal record^63,64^ but when it resumes in the late Oligocene mystacinids were widespread, occurring in deposits in both central and northern Australia^27^. In New Zealand’s oldest terrestrial mammal-bearing deposit, in 19–16 Ma sediments of the lower Bannockburn Formation near St Bathans, mystacinids are present and there is evidence that long-term ecological associations between *Mystacina* and its arthropod prey and roost trees and food plants were already established^30^.

If *Vulcanops* is a mystacinid, as we suggest here, it brings the number of representatives of this bat family in the Miocene St Bathans fauna to four^28,30^. In Australia, at least another four mystacinid species, all in the genus *Icarops*, are recorded from Oligocene to Miocene deposits in South Australia, Queensland and the Northern Territory, with two species co-occurring in some Queensland deposits^27^. In our total evidence analysis (Fig. 3), *Vulcanops* forms a clade with *Mystacina* species, with *Icarops* species paraphyletic relative to *Vulcanops* + *Mystacina*; this arrangement is congruent with a single origin of New Zealand mystacinids from an Australian source, but the topology receives only weak support.

A striking feature distinguishing the dentition of *Vulcanops* from previously known mystacinids (*Mystacina* spp. and *Icarops* spp.) is the presence of a large hypocone on its upper molars (Fig. 2). This structure is similar to that found in neotropical noctilionoids (phyllostomids and mormoopids). In that speciose group, it appears to have evolved multiple times^43^, but it is otherwise uncommon (and particularly rare on M3) in bats with a dilambdodont dentition. Outside Noctilionoidea, a large bulbous hypocone also occurs in the late Eocene Egyptian bat *Aegyptonycteris knightae* Simmons, Seiffert & Gunnell, 2016^65^. The latter is known only from its dilambdodont M2-3 and is the only member of its family whose relationships to other bats are unknown^65^. This large fossil bat differs significantly from *Vulcanops* in that its M2-3 also have a large conule at the base of the metacone and an ectostyle on the buccal margin, two features unknown in other bat families, living or extinct^65^. Among mammals, a hypocone increases occlusal area, effectively doubling the tooth surface devoted to processing food^66^. It is strongly correlated with a less strictly carnivorous diet, often involving an increase in plant consumption^66^. In *Vulcanops*, a long, broad, deep talonid on m1-2, low curved postcristid (=posthypocristid), cristid obliqua lacking carnassial notches, and long broad protocone on M1-2 are also horizontal shearing adaptations associated with a relatively more herbivorous diet. At the same time, elongation of the molar crests as also seen in *Vulcanops* (postmetacrista on M1-2 twice length of preparacrista, shallow ectoloph, open angle of m1-3 trigonids, cristid obliqua meeting trigonid buccal to centre of crown) are adaptations for vertical shearing, possibly indicating relatively more flesh eating.

As body size increases in bats, species with dilambdodont molars often include small vertebrates in their diets^65–70^. The presence of a well-developed hypocone in the ~40 g *Vulcanops*, however, argues against a strictly carnivorous diet. A tall, rounded hypocone is absent in flesh-eating bats (e.g. nycterids, megadermatids and phyllostomines *Vampyrum* and *Trachops*;^71^), although a crestiform hypocone is present in fish-eating noctilionids (*Noctilio* spp.) and is similar to the condition seen in some specimens of *Vulcanops* (e.g. CM 2013.18.916; Fig. 2a). Other aspects of *Noctilio* teeth that are possibly adaptations for piscivory (e.g. the discontinuous centrocrista of M1-2, in which the central blades reach the buccal margin of the crown, and the cristid obliqua of m1-2, which extends to the lingual margin of the crown) are very different from those of *Vulcanops*. The latter’s dentition, and diet, was perhaps most similar to some phyllostomines that consume invertebrates, nectar, fruit, flowers, as well as small vertebrates (e.g. the large-bodied omnivorous *Phyllostomus hastatus*;^65^).

New Zealand’s Recent *Mystacina* species also have very broad, omnivorous diets consisting of nectar, pollen, fruit, flowers, and flying and terrestrial arthropods, but are not known to hunt small vertebrates^72,73^. However, *Vulcanops* exhibits several dental apomorphies, such as a large, blunt hypocone and long, broad, deep talonid, that are lacking in *Mystacina* species (as well as in Australia’s extinct *Icarops* species) and suggest additional feeding capabilities in this extinct bat. No other extant or extinct bat known from the Australasian region has similar dental features. If a large blunt hypocone is indicative of increased herbivory in bats, as argued above (see also^65^), this may provide evidence for the wider adoption, both geographically and taxonomically, of phytophagy in noctilionoid bats by the early Miocene^74^. It may also have relevance to phylogenetic reconstructions of the ancestral diet in Noctilionoidea and its constituent families^17,43,74–77^.

There is some evidence from dental remains that Australia’s extinct *Icarops* species were more insectivorous than New Zealand’s omnivorous extant and extinct *Mystacina* species^27,30^. The derived features present in the dentition of *Vulcanops* that are absent in other mystacinids signal a further shift in diet. This could reflect exploitation of new, abundant and/or underutilized food resources in New Zealand compared with Australia where omnivorous peramelemorphian (bandicoot) and phalangeridan (possum and kangaroo) marsupials were morphologically diverse, speciose and abundant in forest ecosystems shared with mystacinids^27,64^. Baker *et al*.^76^ have argued that the adaptive radiation of feeding strategies seen in phyllostomid noctilionoids – the most radical derived from a common ancestor for any monophyletic group of mammals – was triggered by the dietary inclusion of plant material in addition to insects, in concert with new environmental opportunities in Oligo-Miocene South America.

The large body size (~40 g) estimated for the early Miocene New Zealand mystacinids *Vulcanops jennyworthyae* and *Mystacina miocenalis* Hand, Lee, Worthy & Archer, 2015^30^ is notable compared with other extant and extinct mystacinids (Table 2), and especially given that the ancestral body mass for noctilionoids and the *Mystacina* lineage has been estimated at ~10–14 g^78^. The evolution of relatively large size in certain bat lineages has been associated with ecological release from the biophysical constraints imposed by flight and echolocation during aerial insectivory, and occurs in lineages exhibiting divergent dietary and behavioural specializations such as frugivory (e.g. pteropodids) or gleaning and perch-hunting behaviour in extreme insectivory and animalivory (e.g. megadermatids, noctilionids)^78^. That some mystacinids have reached notably large sizes may be another example of this evolutionary trend. Mystacinids are renowned for their peculiar walking habits which enable them to exploit an exceptionally broad range of plant and animal resources^72^, including ground-flowering plants and large invertebrate prey that they can pursue on foot.

In early Miocene New Zealand, *V. jennyworthyae* was part of a diverse faunal community living in semitropical to warm-temperate Gondwanan rainforest on the shores of the vast 5000 sq km Manuherikia palaeolake^30,79,80^. A number of distinctive vertebrate taxa present in the early Miocene St Bathans assemblage, like *Vulcanops*, disappeared sometime before the late Pleistocene. These include crocodilians, terrestrial turtles, flamingo-like palaelodids, swiftlets, several pigeon, parrot and shorebird lineages and non-volant mammals (e.g.^8,9,31–33,36,37^). Most of these were probably warm-adapted species^8,9,81^. After the middle Miocene, global climate change^59^ brought colder and drier conditions to New Zealand, with significant changes to vegetation and palaeoenvironments^80,82^. It is possible that this general cooling and drying trend also drove extinction of the *Vulcanops* lineage, and overall loss in mystacinid diversity over time. In Australia, the *Icarops* lineage also went extinct, sometime after the late middle Miocene, with Mystacinidae being the only one of eight crown bat families known to have become extinct on that continent^62^. The reasons for this remain unclear, in part because the later Miocene and Pliocene Australian mammal record is too poor to pinpoint the time of their disappearance^62,64^.

## Methods

Stratigraphic nomenclature for the St Bathans region follows Schwarzhans *et al*.^35^. Dental terminology follows Hand *et al*.^27^ and Dávalos *et al*. (ref.^43^: MorphoBank P891). Case denotes upper (e.g. M1) and lower (e.g. m1) teeth. The prefix CM refers to specimens held in the fossil collections of the Canterbury Museum, Christchurch, New Zealand; NMNZ to the Museum of New Zealand Te Papa Tongarewa, Wellington. Species examined in this study are listed in Supplementary Information online (see also MorphoBank P2737).

To assess its likely phylogenetic affinities, *Vulcanops* was added to a large morphological character matrix (MorphoBank Project 2737; http://morphobank.org/permalink/?P2737) comprising 292 dental characters scored for 45 yangochiropterans (35 extant and 10 fossil species) plus 2 yinpterochiropteran outgroup taxa. *Vulcanops* could be scored for 143 of 292 characters, rendering it 49% complete. 112 characters representing plausible morphoclines were specified as ordered. We also created a total evidence matrix by combining the morphological dataset with the molecular dataset of Amador *et al.* ^20^. This comprises DNA sequence data from five nuclear genes [dentin matrix protein 1 (*DMP1*), recombination activating protein 1 (*RAG1*), recombination activating protein 2 (*RAG2*), exon 11 of the breast cancer susceptibility protein 1 (*BRCA1*) and exon 28 of the von Willebrand factor (*VWF*)] and four mitochondrial genes [cytochrome b (*MT-CYB*), NADH:ubiquinone oxidoreductase core subunit 1 (*MT-ND1*), and 12 S (*MT-RNR1*) and 16 S (*MT-RNR2*) rRNAs]. We maintained Amador *et al*.’s^20^ alignment, but deleted the third codon position of MT-CYB, because this partition showed the greatest evidence of compositional heterogeneity (calculated using BaCoCa; Kück & Struck^83^), leaving 11.1 kb of sequence data. We then pruned this modified alignment down to the extant taxa present in our morphological matrix.

The total evidence matrix was analysed using an undated Bayesian approach in MrBayes 3.2.6^84^. First, PartitionFinder 2.1.1^85^ was used to select an appropriate partitioning scheme and set of models for the molecular data, assuming linked branch lengths, and using the “greedy” algorithm and AICc for model selection; only models implemented by MrBayes were tested. The morphological data was assigned the Mk model of Lewis^86^, assuming that variable characters had been scored, and with a gamma distribution with four rate categories to model rate heterogeneity among the morphological characters. The MrBayes analysis comprised four runs of four chains (three “heated,” one “cold”), sampling trees every 5000 generations. The analysis was run for 5 × 10^6^ generations, with the first 25% of sampled trees discarded as burn-in; the post-burn-in trees were summarised using 50% majority rule consensus, with Bayesian posterior probabilities as support values.

To estimate body mass in extinct bats, Gunnell *et al*.^38^ developed a set of algorithms based on dental, skeletal and weight measurements in 1,160 extant bats from eight families. We used these equations, and the proxies of upper first molar (M1) area, lower first molar (m1) area, and diameter of mid-shaft humerus, to estimate the body mass of eight of the ten known extinct and extant mystacinid taxa (Table 2).

The morphological datasets generated or analysed during this study are included in this published article’s tables or are available in the MorphoBank repository as Project 2737 (http://morphobank.org/permalink/?P2737).

### Nomenclatural Act

This published work and the nomenclatural acts it contains have been registered in ZooBank, the proposed online registration system for the International Code of Zoological Nomenclature. The ZooBank life science identifiers can be resolved and the associated information viewed by appending the life science identifiers to the prefix http://zoobank.org/. The life science identifier for this publication is 13BDAB9F-4BC3-4711-A331-4E883DE52DC2, for Vulcanops is 498FA8AA-7DAF-4703-94F3-02931CE7F85F, and for V. jennyworthyae is 3A625804-F490-46F6-BDA0-C93A6523EE6D.

## Electronic supplementary material

Supplementary information
