# Supplementary material for: A new, large-bodied omnivorous bat (Noctilionoidea: Mystacinidae) reveals lost morphological and ecological diversity since the Miocene in New Zealand
Source: Sci Rep. 2018 Jan 10;8:235. doi: 10.1038/s41598-017-18403-w (PMC5762892; doi:10.1038/s41598-017-18403-w)
Supplement: Supplementary file 1 — Supplementary information [file 41598_2017_18403_MOESM1_ESM.pdf]

SUPPLEMENTARY INFORMATION

A new, large-bodied omnivorous bat (Noctilionoidea: Mystacinidae) reveals lost morphological and ecological diversity since the Miocene in New Zealand

Suzanne J. Hand, Robin M. D. Beck, Michael Archer, Nancy B. Simmons, Gregg F. Gunnell, R. Paul Scofield, Alan J. D. Tennyson, Vanesa L. De Pietri, Steven W. Salisbury & Trevor H. Worthy

List of comparative bat material used in this study

Institutional abbreviations: AM, Australian Museum, Sydney; AMNH, American Museum of Natural History Mammal Collection, New York; AR, Vertebrate Palaeontology Collection, University of New South Wales, Sydney; CGM, Cairo (= Egyptian) Geological Museum, Egypt; CM, Canterbury Museum, Christchurch; FMNH, Field Museum of Natural History, Chicago; MNHN, Muséum national d'Histoire naturelle, Paris; NMNZ, Museum of New Zealand Te Papa Tongarewa, Wellington; QM, Queensland Museum, Brisbane, Australia; SMF, Naturmuseum Senckenberg, Frankfurt; UF, University of Florida, Gainesville, USA; USNM, National Museum Natural History, Smithsonian Institution, Washington D. C.; YPM, Yale Peabody Museum, New Haven, Connecticut, USA.

Fossil specimens: Myzopodidae: *Phasmatonycteris butleri* CGM 83761, Fayum Quarry BQ-2, 23 m level, Birket Qarun Formation, Fayum Depression, Egypt, Late Eocene; *P. phiomensis* YPM 24198, Fayum Quarry I, 242 m level, Upper Gebel Qatrani Formation, Fayum Depression, Egypt, Early Oligocene. Mystacinidae: *Mystacina robusta* NMNZ S.35205, Exhale Air Cave, Ellis Basin, Mt Arthur, Nelson, New Zealand, Holocene; *M. tuberculata*

NMNZ S.32400-parts 1–20, Predator Cave, Takaka Hill, Nelson, New Zealand, Holocene; *M. miocenalis* CM 2013.18.381, NMNZ.S52355, and Mystacinidae indet. 1 & 2, NMNZ S.41867, S.42215, S.44260, S.51739, S.51742, S.52083, S.52401, S.52402, S.52920, Manuherikia River section, Bannockburn Formation, Home Hills Station, St Bathans, Central Otago, New Zealand, Early Miocene; *Icarops aenae* QM F30583–4, F30586, F24509, F30573-4 and *I. paradox* QM F30580–3, F30590, F31561, Riversleigh World Heritage Area, Queensland, Australia, Early Miocene. Phyllostomidae: *Notonycteris magdalenensis* UCMP 39960, 39962, 39963, IGM 252865, 252973, IGM-KU 90-C01, Villavieja Formation, Huila Department, La Venta, Colombia, Middle Miocene; *N. sucharadeus* IGM-KU 9305, IGM 252869, Villavieja Formation, Huila Department, La Venta, Colombia, Middle Miocene. Noctilionoid family indet.: *Speonycteris aurantiadens* UF 240000, University of Florida locality Brooksfield 2, Hernando County, Florida, USA, Late Oligocene; *S. naturalis* UF121717 University of Florida locality I-75, Alachua County, Florida, USA, Early Oligocene.

Modern comparative specimens (and see also MorphoBank Project 2737): Craseonycteridae: *Craseonycteris thonglongyai* SMF 54513. Emballonuridae: *Emballonura atrata* FMNH 176360; *Saccolaimus saccolaimus* AM M3491; *Saccopteryx bilineata* AMNH M267842; *Taphozous georgianus* AR20502. Furipteridae: *Furipteris horrens* AMNH M267213. Hipposideridae: *Anthops ornatus* AM M5831; *Aselliscus tricuspidatus* SMF 24745; *Hipposideros bicolor* AM M9231; *Hipposideros diadema* AR5194. Megadermatidae: *Lavia frons* AR20507; *Macroderma gigas* AR20505. Miniopteridae: *Miniopterus orianae* AR1868; *M. australis* AM M5556. Molossidae: *Cheiromeles parvidens* AMNH M241942; *Chaerephon jobensis* QM JM5161; *Chaerephon plicatus* AMNH M107932; *Cynomops planirostris* AMNH M234455; *Eumops auripendulus* AMNH M248212; *Molossus molossus* AMNH M267243; *Mops brachypterus* AMNH M241062; *Mormopterus beccarii* AM M8509; *Mormopterus planiceps* AR20503; *Myopterus daubentonii* AMNH M48855; *Promops nasutus* AMNH

M184648; *Tadarida australis* AM M7910. Mormoopidae: *Mormoops blainvillei* AMNH M238144; *Pteronotus davyi* AMNH M204961. Mystacinidae: *Mystacina tuberculata* NMNZ LM1231. Myzopodidae: *Myzopoda aurita* USNM 448932; MNHN 1907.618. Natalidae: *Natalus stramineus* USNM 362103. Noctilionidae: *Noctilio albiventris* USNM 390592. Nycteridae: *Nycteris capensis* AM M6071; *Nycteris javanica* AMNH M103269. Phyllostomidae: *Anoura geoffroyi* AMNH M264935; *Artibeus jamaicensis* AR21504; *Desmodus rotundus* AMNH M248941; *Glossophaga soricina* AMNH M230206; *Micronycteris hirsuta* AMNH M267860; *Phyllostomus hastatus* AMNH M267901; *Tonatia saurophila* AMNH M267912; *Trachops cirrhosus* AMNH M266081. Pteropodidae: *Balionycteris maculata* AMNH M233970; *Nyctimene robinsoni* AR17520; *Pteropus scapulatus* AR4764, AR4764A; *Rousettus amplexicaudatus* USNM 78616. Rhinolophidae: *Rhinolophus megaphyllus* AR1655. Rhinonycteridae: *Rhinonicteris aurantia* AR20501. Rhinopomatidae: *Rhinopoma hardwickei* AR21506. Thyropteridae: *Thyroptera tricolor* AMNH M266356. Vespertilionidae: *Antrozous pallidus* USNM 564036; *Chalinolobus morio* AR1653; *Chalinolobus gouldii* QM JM5061; *Eptesicus fuscus* AMNH M139513; *Falsistrellus tasmaniensis* AM M3360; *Kerivoula papillosa* AMNH M247576; *Myotis macropus* AM M5453; *Nyctophilus gouldi* AM M12548; *Scoteanax ruepelli* AM M5163; *Scotorepens orion* AM M5163; *Vespadelus vulturnus* AM M11413.

Description of specimens referred to *Vulcanops jennyworthyae* gen. et sp. nov.

The holotype (CM 2013.18.790) is a dentary preserving m2-3. The specimen is broken anteriorly through the posterior root of m1, and posterodorsally such that the mandibular foramen and part of the masseteric fossa are preserved but not the ascending ramus (coronoid process), mandibular condyle or angular process. The horizontal (mandibular) ramus has a

straight ventral margin. The mandibular foramen is large and elongate, opening posterodorsally well below the level of the tooth row.

The m1 (NMNZ S.42876, NMNZ S.52078) has two roots and five cusps. The trigonid is shorter and conspicuously narrower than the talonid. The protoconid, hypoconid and entoconid are the dominant cusps in terms of both height and volume. The protoconid is taller than the hypoconid, which is taller than the entoconid, which is taller than the metaconid and paraconid. The hypoconulid is a small cingular cusp. The protoconid and hypoconid have buccally rounded, smooth flanks; the protoconid shows more wear than the other cusps. The cristid obliqua, in occlusal view, is curved and contacts the trigonid at a point conspicuously buccal to the midpoint between the protoconid and metaconid. In lateral view, there is a slight buccal inflexion in the cristid obliqua close to the trigonid. The postcristid extends from the hypoconid directly to the entoconid, almost perpendicular to the axis of the tooth row, isolating the hypoconulid and thereby exhibiting the myotodont condition. The greatly bowed curve in the hypocristid, best seen in posterior view, occurs approximately midway between the hypoconid and entoconid. The angle between the para- and protocristids is wide, at ~75°. The cristid obliqua and paracristid are subparallel. A small crest extends between the metaconid and paraconid. A steeply dipping, curved and inflected entocristid attenuates at the base of the metaconid without completely closing the talonid basin lingually. There is a non-sinuous, continuous anterior, buccal and posterior cingulid: it is narrow buccally, particularly around the face of the hypoconid. The posterior portion of the cingulid is wider than the anterior cingulid.

The m2 (CM 2013.18.790) is described in so far as it differs from m1. The trigonid is relatively wider. The entoconid is taller than the hypoconid, metaconid and paraconid. All portions of the continuous cingulid are of similar width.

The m3 (CM AV 2013.18.790) is shorter and narrower than m1-2. The trigonid is wider than the talonid. The talonid cusps (including the entoconid) are lower than the trigonid cusps. The hypoconulid is smaller. The hypoconid is less buccally rounded. The cristid obliqua contacts the trigonid at a point closer to the junction of the components of the protocristid.

Referral of the upper teeth (described below) to the same taxon as the lower dentition is based on their provenance, appropriate size and evident occlusal relationships. Representatives of all known tooth positions (m1, m2, m3, M1, M2, M3) were recovered from Bed HH1a, a 5–10 cm thick sandy conglomerate in the Manuherikia River section.

M1 (CM 2013.18.916, NMNZ S.44071, S.51461, CM 2013.18.1) is approximately as wide as long. The metacone is more massive than the paracone but topographically similar in height. The buccal basin of the paracone is very shallow, the basin of the metacone slightly deeper. The protocone is the most voluminous cusp. The apex of the protocone is just posterolingual to the paracone apex. The ectoloph is W-shaped, with the centrocrista reaching the buccal margin of the tooth. A conical parastyle is variably developed. A mesostyle is present but is not cuspidate; the metastyle is pronounced. The buccal margin of the tooth is relatively straight (and oblique with respect to the paracone), with a very shallow postectoflexus. An indistinct, variably present ectocingulum runs between the parastyle and mesostyle and/or mesostyle and metastyle. The preparacrista is shorter than the postparacrista, which is shorter than the premetacrista, which in turn is shorter than the postmetacrista. In CM 2013.18.916, the latter is especially elongated (twice postparacrista length). The pre- and postparacrista meet at an angle just over 90°, the pre- and postmetacristae at ~80° and the postparacrista and premetacrista ~60°. There is no clear paraloph or metaloph. The protofossa is deep and broad, and opens posteriorly. The preprotocrista continues buccally as a narrow paracingulum. The posterobuccally directed postprotocrista does not reach the metacone

base, and terminates in a metaconule. An indistinct cingulum extends from a point posterior to the protocone base to the metastyle, but it does not connect to the cingulum enclosing the well-developed heel. The cingulum is massively swollen lingually (near the protocone), forming a hypocone. The hypocone itself is cuspidate or bulbous rather than crested. The heel is long (representing half the lingual length of the crown), relatively wide (40% the width of the crown), and directed posterolingually. An indentation in the lingual border of the crown separating the hypocone from the protocone base is variably developed. There is no cingulum around the protocone. The tooth has three roots; the protocone root is long (anteroposteriorly) and broad, the metacone root large and triangular, and the paracone root smaller and circular.

NMNZ S.50383 and NMNZ S.51746 are upper molars similar in size and overall morphology to NMNZ S.44071, NMNZ S.51461, CM 2013.18.1 and CM 2013.18.916. They differ from those molars in features typically distinguishing M2 from M1 in bats, and are described in so far as they differ from M1. M2 is wider (especially anteriorly) and has a larger paracone and deeper paracone basin. The preparacrista is only just shorter than the subequal postparacrista and premetacrista and longer postmetacrista. The buccal cingulum is more distinct. There is no paraloph or metaloph. The cingulum enclosing the well-developed heel forms a tall hypocone anterolingually (near the protocone), as in M1. The paracone and metacone root are more similar in size, the protocone root being again very long and broad.

M3 (NMNZ S.52400, S.52351, S.50384) is wider than long; it is shorter and narrower than M2. It retains a metacone, subequal in height to the paracone. The preparacrista is longer than the subequal postparacrista and premetacrista, and bears a wear facet from the m3 protocristid. The paracingulum is narrow as in M1-2, and the protofossa broad and deep. There is no paraloph. The posterobuccally directed postprotocrista meets the base of the metacone, enclosing the protofossa. A buccal cingulum extends from parastyle to metacone,

attenuating slightly at the mesostyle. There is no anterolingual cingulum, but the heel-like posterolingual cingulum bears a distinct cuspule (hypocone). The protocone root and lingually displaced metacone root are similar in size and probably larger than the broken paracone root.

SUPPLEMENTARY DATASET 1

TABLE S1. Taxon-character matrix comprising 292 dental characters scored for 45 yangochiropterans (35 extant and 10 fossil species) plus 2 yinpterochiropteran outgroup taxa is available in the MorphoBank repository as Project 2737 (http://morphobank.org/permalink/?P2737)

SUPPLEMENTARY DATASET 2

TABLE S2. List of synapomorphies for nodes under Accelerated Transformation (ACCTRAN) and Delayed Transformation (DELTRAN) from Bayesian analysis of total evidence matrix.

1. Bayesian analysis of total evidence data - synapomorphies under Accelerated Transformation (ACCTRAN)

```
P A U P *
Version 4.0a152 for 32-bit Microsoft Windows (built on Jan 26 2017 at 21:47:42)
Thu Jun 22 09:33:46 2017

   ----------------------------NOTICE----------------------------
      This is an alpha-test version that is still changing rapidly.
      It will expire on 1 Jul 2017.

      Please report bugs to david.swofford@duke.edu
     --------------------------------------------------------------


paup> set opt=acctran;
```

```
paup> describe /apolist;

Tree description:

  Unrooted tree(s) rooted using outgroup method
  Optimality criterion = parsimony
    Character-status summary:
      Of 292 total characters:
        112 characters are of type 'ord' (Wagner)
        180 characters are of type 'unord'
        All characters have equal weight
        37 characters are constant
        22 variable characters are parsimony-uninformative
        Number of parsimony-informative characters = 233
    Gaps are treated as "missing"
    Multistate taxa interpreted as uncertainty
    Character-state optimization: Accelerated transformation (ACCTRAN)

Tree 1 ("con 50 majrule") (rooted using user-specified outgroup)

Tree length = 1689
Consistency index (CI) = 0.2404
Homoplasy index (HI) = 0.7596
CI excluding uninformative characters = 0.2294
HI excluding uninformative characters = 0.7706
Retention index (RI) = 0.4491
Rescaled consistency index (RC) = 0.1080

                                    /----- Speonycteris aurantiadens
                               /---88
                               |    \----- Micronycteris hirsuta
              /---------------------------87
              |                \---------- Mimon bennettii
              |
              |                     /----- Macrotus californicus
              +-------------------------------71
              |                     \----- Macrotus waterhousii
              |
              |                     /----- Desmodus rotundus
              |                /---72
          /---86               |    \----- Diaemus youngi
          |   |   /----------------------73
          |   |   |            \---------- Diphylla ecaudata
          |   |   |
          |   |   |           /--------------- Lophostoma evotis
          |   |   |           |
          |   |   |           +--------------- Phyllostomus hastatus hastatus
          |   |   |       /--76
          |   |   |       |   |   /---------- Phylloderma stenops stenops
          |   |   |       |   |   |
```

```
                |   \---85         /---77     \---75      /----- Notonycteris magdalenensis
                |        |        |    |      \---74
                |        |        |    |          \----- Notonycteris sucharadeus
                |        |   /---78      |
                |        |   |    |    \------------------- Tonatia saurophila saurophila
                |        |   |    |
            /---70         |  |      \------------------------ Trachops cirrhosus cirrhosus
            |    |        |   |
            |    |        |   |              /---------- Anoura geoffroyi peruana
            |    |       \---84                 |
            |    |           |           /---80     /----- Phyllonycteris aphylla
            |    |           |            |   \---79
            |    |           |        /--81         \----- Leptonycteris yerbabuenae
            |    |           |        |   |
            |    |           \--------83     \--------------- Lonchophylla thomasi
            |    |                    |
            |    |                    |          /----- Carollia perspicillata
            |    |                   \------------82
            |    |                              \----- Artibeus jamaicensis
        /--69      |
         |   |    |                          /---------- Mormoops blainvillei
         |   |    |                          |
         |   |    \--------------------------------49       /----- Pteronotus davyi
         |   |                              \---48
         |   |                                  \----- Pteronotus parnellii
         |   |
         |   |                                  /----- Noctilio albiventris
         |   |                              /---50
         |   |                               |   \----- Noctilio leporinus
         |   |                          /---52
         |   |                          |    |    /----- Furipterus horrens
         |   |                          |    \---51
         |  \--------------------------------54            \----- Amorphochilus schnablii
    /---68                                |
     |    |                             |        /----- Thyroptera discifera
     |    |                             \--------53
     |    |                                     \----- Thyroptera tricolor
     |    |
     |    |                                     /----- Vulcanops worthyae
     |    |                                     |
     |    |                                     +----- Mystacina robusta
     |    |                                 /---55
     |    |                                  |   +----- Mystacina tuberculata
/---67      |                                  |    |
|    |    |                              /---56     \----- Mystacina miocenalis
|    |    |                               |   |
|    |    \------------------------------------58        \---------- Icarops paradox
|    |                                  |
|    |                                  |        /----- Icarops aenae
|    |                                  \--------57
|    |                                          \----- Icarops breviceps
```

```
    |   |
    |   |                                    /--------- Natalus stramineus
    |   |                                    |
/---64   \-------------------------------------------66    /----- Chalinolobus gouldii
|   |                                        \---65
|   |                                            \----- Chaerephon jobensis
|   |
|   |                                            /----- Myzopoda aurita
|   |                                        /---59
|   |                                        |   \----- Myzopoda schliemanni
|   |                                    /---60
63  |                                /--61   \---------- Phasmatonycteris butleri
|   |                                |   |
|   \-------------------------------------62   \-------------- Phasmatonycteris phiomensis
|                                    |
|                                    \----------------- Saccopteryx bilineata
|
+--------------------------------------------------------------- Hipposideros commersoni
|
\--------------------------------------------------------------- Pteropus scapulatus
```

Apomorphy lists:

```
         Branch              Character  Steps     CI   Change
-----------------------------------------------------------------
       node_63 --> node_64        5          1   0.273  1 ==> 0
                          16          1   0.222  0 --> 1
                          18          1   0.167  0 --> 1
                          29          2   0.214  2 ==> 0
                          50          2   0.400  0 --> 2
                          61          1   0.154  1 --> 0
                          102         1   0.200  1 --> 2
                          105         1   0.133  0 --> 1
                          112         1   0.125  0 --> 1
                          120         1   0.143  0 --> 1
                          123         1   0.167  0 --> 1
                          129         1   0.154  0 --> 1
                          136         1   0.500  0 --> 1
                          153         1   0.222  2 --> 1
                          154         1   0.143  1 ==> 0
                          155         1   0.200  1 --> 0
                          158         1   0.200  0 ==> 1
                          186         2   0.158  1 --> 3
                          187         2   0.158  0 --> 2
                          194         1   0.143  1 ==> 2
                          206         1   0.286  1 --> 2
                          210         1   0.200  1 ==> 2
                          214         1   0.333  2 --> 3
                          233         1   0.286  0 --> 1
                          242         2   0.333  0 --> 2
                          267         1   0.154  1 --> 0
```

	274	1	0.250	1 --> 2
	275	1	0.333	1 --> 2
node_64 --> node_67	9	1	0.250	0 --> 1
	11	1	0.250	0 ==> 1
	13	1	0.273	1 --> 2
	40	1	0.231	2 --> 3
	52	1	1.000	0 --> 1
	55	1	0.167	1 ==> 0
	97	1	0.125	0 --> 1
	98	1	0.286	0 --> 4
	192	1	0.214	1 --> 3
	215	1	0.143	0 --> 1
	278	1	0.091	1 --> 0
node_67 --> node_68	6	2	1.000	0 --> 2
	12	1	0.455	1 ==> 0
	16	1	0.222	1 --> 0
	74	1	0.200	1 --> 0
	84	1	0.200	2 --> 0
	117	1	0.167	0 --> 1
	155	1	0.200	0 --> 1
	156	1	0.500	1 --> 2
	200	1	0.500	0 --> 1
	220	1	0.111	0 --> 1
	260	1	0.250	0 --> 1
	277	1	0.091	0 --> 1
node_68 --> node_69	1	1	0.100	0 --> 1
	24	1	0.100	1 --> 0
	29	1	0.214	0 --> 1
	34	1	0.158	1 ==> 0
	36	1	0.333	0 --> 1
	60	1	0.158	2 --> 1
	61	1	0.154	0 --> 1
	106	1	0.107	0 --> 1
	164	1	0.222	1 ==> 2
	184	1	0.143	1 --> 0
	196	1	0.200	0 --> 1
	202	1	0.182	0 --> 1
	275	1	0.333	2 --> 1
	278	1	0.091	0 --> 1
node_69 --> node_70	5	1	0.273	0 --> 1
	13	1	0.273	2 ==> 3
	18	1	0.167	1 --> 0
	29	1	0.214	1 ==> 2
	41	1	1.000	0 --> 1
	42	1	1.000	1 --> 0
	59	1	0.125	1 --> 0
	60	1	0.158	1 ==> 0
	67	1	0.143	1 --> 2
	69	1	0.154	1 --> 0
	106	1	0.107	1 --> 2
	110	1	0.143	2 ==> 1

	111	1	0.111	0 --> 1
	127	1	0.138	2 ==> 1
	128	1	0.143	0 ==> 1
	158	1	0.200	1 --> 0
	173	2	0.286	2 ==> 0
	215	1	0.143	1 ==> 0
	216	1	1.000	1 --> 0
	234	1	0.200	1 ==> 0
	241	1	0.250	0 ==> 1
node_70 --> node_86	19	1	0.200	0 --> 1
	23	1	0.133	1 --> 0
	49	1	0.286	0 ==> 1
	54	1	0.200	0 ==> 1
	78	1	0.200	1 --> 0
	93	1	0.200	0 --> 1
	97	1	0.125	1 --> 0
	131	1	0.444	1 ==> 2
	139	4	0.227	1 ==> 5
	140	2	0.222	0 ==> 2
	152	1	0.333	1 ==> 0
	167	1	0.154	2 --> 1
	263	1	0.222	0 ==> 1
	267	1	0.154	0 --> 1
	271	1	0.200	1 --> 0
node_86 --> node_87	23	1	0.133	0 --> 1
	25	1	0.200	2 ==> 3
	51	1	0.167	0 ==> 1
	59	1	0.125	0 --> 1
	71	1	0.111	2 ==> 1
	83	1	0.200	0 ==> 1
	108	1	0.333	0 ==> 1
	124	1	0.333	0 ==> 1
	143	1	0.200	0 ==> 1
	153	1	0.222	1 --> 0
	165	1	0.125	1 --> 2
	184	1	0.143	0 --> 1
	203	1	0.250	1 --> 0
	210	1	0.200	2 --> 1
	260	1	0.250	1 ==> 0
node_87 --> node_88	24	1	0.100	0 --> 1
	34	1	0.158	0 --> 1
	38	1	0.250	0 ==> 1
	53	1	0.125	0 --> 1
	55	1	0.167	0 --> 1
	57	1	0.286	0 --> 1
	60	2	0.158	0 --> 2
	63	2	0.125	0 ==> 2
	87	1	0.250	0 --> 1
	98	1	0.286	4 --> 3
	103	1	0.200	1 --> 2
	106	1	0.107	2 --> 3

112 1 0.125 1 --> 2
135 1 0.200 0 --> 1
146 1 0.250 1 --> 0
161 1 0.167 0 --> 1
164 1 0.222 2 ==> 1
253 1 0.278 1 --> 0
node_88 --> Speonycteris aurantiadens 23 1 0.133 1 ==> 2
27 1 1.000 0 ==> 1
165 1 0.125 2 --> 1
167 1 0.154 1 --> 2
176 1 0.400 0 ==> 1
186 1 0.158 3 ==> 4
187 1 0.158 2 ==> 3
192 1 0.214 3 ==> 1
202 1 0.182 1 ==> 2
210 1 0.200 1 --> 2
220 1 0.111 1 ==> 0
221 1 0.143 1 ==> 0
237 1 0.333 1 ==> 0
252 1 0.182 0 ==> 1
264 1 0.200 0 ==> 1
node_88 --> Micronycteris hirsuta 26 1 0.250 0 ==> 1
31 1 0.286 1 ==> 2
61 1 0.154 1 ==> 0
64 1 0.667 1 ==> 2
162 1 0.364 2 ==> 1
172 1 0.167 0 ==> 1
203 1 0.250 0 --> 1
207 1 0.100 0 --> 1
221 1 0.143 1 ==> 2
253 1 0.278 0 --> 2
263 1 0.222 1 ==> 0
node_87 --> Mimon bennettii 1 1 0.100 1 ==> 0
16 1 0.222 0 --> 1
18 1 0.167 0 ==> 1
47 1 0.200 1 ==> 0
49 1 0.286 1 ==> 0
54 1 0.200 1 ==> 0
84 1 0.200 0 --> 2
93 1 0.200 1 --> 0
105 1 0.133 1 ==> 0
106 2 0.107 2 ==> 0
111 1 0.111 1 ==> 0
117 1 0.167 1 ==> 0
120 1 0.143 1 ==> 2
128 1 0.143 1 ==> 0
148 2 0.143 2 ==> 0
150 1 0.200 0 ==> 1
167 1 0.154 1 ==> 0
174 1 0.167 0 ==> 1
196 1 0.200 1 ==> 0

223 1 0.222 1 ==> 2
226 1 0.111 0 ==> 1
232 1 0.200 1 ==> 0
234 1 0.200 0 ==> 1
269 1 0.222 0 ==> 1
271 1 0.200 0 --> 1
278 1 0.091 1 ==> 0
node_86 --> node_71 37 1 0.200 1 ==> 0
53 1 0.125 0 ==> 1
56 1 0.250 0 ==> 1
63 1 0.125 0 ==> 1
84 1 0.200 0 --> 2
98 1 0.286 4 ==> 2
105 1 0.133 1 ==> 2
106 1 0.107 2 ==> 3
118 1 0.375 4 ==> 2
132 1 0.250 0 ==> 1
146 1 0.250 1 ==> 0
161 1 0.167 0 ==> 1
167 1 0.154 1 --> 2
186 2 0.158 3 ==> 1
207 1 0.100 0 --> 1
208 1 0.250 0 ==> 1
215 1 0.143 0 ==> 1
217 1 0.286 2 ==> 1
220 1 0.111 1 ==> 2
223 1 0.222 1 ==> 2
235 1 0.250 1 ==> 2
236 1 0.143 0 --> 1
237 1 0.333 1 ==> 2
node_71 --> Macrotus californicus 20 1 0.333 0 ==> 1
61 1 0.154 1 ==> 0
78 1 0.200 0 --> 1
112 1 0.125 1 ==> 0
148 1 0.143 2 ==> 1
170 1 0.333 0 ==> 1
192 1 0.214 3 ==> 2
221 1 0.143 1 ==> 2
226 1 0.111 0 ==> 1
236 1 0.143 1 --> 2
250 1 0.222 1 ==> 0
253 1 0.278 1 ==> 0
node_71 --> Macrotus waterhousii 26 1 0.250 0 ==> 1
34 1 0.158 0 ==> 1
38 1 0.250 0 ==> 1
58 1 0.167 2 ==> 1
69 1 0.154 0 --> 1
87 1 0.250 0 ==> 1
123 1 0.167 1 ==> 2
131 1 0.444 2 ==> 3
182 1 0.200 0 ==> 1

185	1	0.250	0 ==> 1	
187	2	0.158	2 ==> 0	
195	1	0.182	0 ==> 1	
206	1	0.286	2 ==> 1	
244	1	0.333	0 ==> 1	
249	1	0.111	0 ==> 1	
250	1	0.222	1 ==> 2	
254	2	0.182	2 ==> 0	
270	1	0.333	0 ==> 1	
node_86 --> node_85	5	1	0.273	1 --> 2
11	1	0.250	1 --> 0	
19	1	0.200	1 --> 0	
31	1	0.286	1 --> 0	
50	1	0.400	2 --> 1	
61	1	0.154	1 --> 2	
69	1	0.154	0 --> 1	
110	1	0.143	1 --> 2	
119	1	0.333	0 --> 1	
127	2	0.138	1 --> 3	
134	1	0.200	1 --> 0	
167	1	0.154	1 --> 0	
175	1	0.143	1 --> 2	
185	2	0.250	0 ==> 2	
187	1	0.158	2 --> 1	
194	2	0.143	2 --> 0	
195	1	0.182	0 --> 2	
220	1	0.111	1 --> 0	
245	2	0.222	0 --> 2	
247	1	0.200	1 --> 0	
252	1	0.182	0 --> 1	
255	1	0.200	1 --> 0	
260	1	0.250	1 --> 2	
264	1	0.200	0 --> 1	
269	1	0.222	0 --> 1	
node_85 --> node_73	5	1	0.273	2 --> 4
12	5	0.455	0 ==> 5	
14	1	0.667	0 --> 2	
17	1	0.200	0 --> 1	
20	1	0.333	0 ==> 1	
21	1	0.125	0 ==> 1	
25	2	0.200	2 ==> 0	
29	1	0.214	2 ==> 3	
38	1	0.250	0 ==> 1	
39	1	0.333	0 ==> 1	
46	1	0.250	0 ==> 1	
58	1	0.167	2 --> 1	
64	1	0.667	1 ==> 0	
69	1	0.154	1 ==> 2	
70	1	0.400	2 ==> 1	
75	1	0.333	0 ==> 1	
98	1	0.286	4 ==> 0	

	101	1	0.500	0 ==> 1
	105	1	0.133	1 --> 0
	110	2	0.143	2 ==> 4
	114	1	0.250	0 ==> 1
	118	1	0.375	4 ==> 0
	127	1	0.138	3 ==> 4
	130	1	0.333	0 ==> 1
	159	1	0.250	0 ==> 1
	160	1	0.111	0 ==> 1
	162	1	0.364	2 ==> 4
	164	2	0.222	2 ==> 0
	165	1	0.125	1 ==> 0
	169	1	0.200	0 ==> 1
	178	1	0.250	0 ==> 1
	186	1	0.158	3 --> 2
	187	1	0.158	1 ==> 0
	189	1	1.000	1 ==> 2
	207	1	0.100	0 --> 1
	217	2	0.286	2 ==> 0
	221	1	0.143	1 ==> 0
	223	1	0.222	1 ==> 0
	227	2	0.286	0 ==> 2
	233	1	0.286	1 ==> 2
	235	1	0.250	1 --> 0
	236	1	0.143	0 ==> 1
	237	1	0.333	1 --> 0
	239	1	1.000	0 ==> 1
	243	2	0.333	0 ==> 2
	251	2	0.333	0 ==> 2
	252	1	0.182	1 ==> 2
	253	1	0.278	1 ==> 5
	259	1	0.125	0 --> 1
	262	3	0.375	0 --> 3
	263	1	0.222	1 --> 2
	264	1	0.200	1 --> 2
	265	1	0.500	0 ==> 1
node_73 --> node_72	15	1	0.250	0 ==> 1
	146	1	0.250	1 --> 0
	149	1	0.333	0 ==> 1
	153	1	0.222	1 ==> 0
	202	1	0.182	1 ==> 0
	204	1	0.500	1 ==> 0
	257	1	1.000	0 ==> 1
node_72 --> Desmodus rotundus	25	1	0.200	0 --> 1
	58	2	0.167	1 ==> 3
	63	1	0.125	0 ==> 1
	67	2	0.143	2 ==> 0
	106	2	0.107	2 ==> 0
	113	1	1.000	0 ==> 1
	154	1	0.143	0 ==> 1
	158	2	0.200	0 ==> 2

186 1 0.158 2 --> 3
220 1 0.111 0 --> 1
251 1 0.333 2 --> 1
node_72 --> Diaemus youngi 70 1 0.400 1 ==> 0
145 1 0.667 0 ==> 3
151 1 0.400 0 ==> 1
210 1 0.200 2 ==> 3
222 1 0.500 0 ==> 1
node_73 --> Diphylla ecaudata 76 1 0.500 0 ==> 1
146 1 0.250 1 ==> 2
152 3 0.333 0 ==> 3
153 1 0.222 1 ==> 2
186 1 0.158 2 ==> 1
node_85 --> node_84 9 1 0.250 1 --> 0
16 1 0.222 0 --> 1
24 1 0.100 0 --> 1
66 1 0.286 0 --> 1
71 1 0.111 2 --> 1
93 1 0.200 1 --> 0
148 1 0.143 2 --> 1
node_84 --> node_78 11 1 0.250 0 --> 1
19 1 0.200 0 --> 1
29 1 0.214 2 ==> 1
47 1 0.200 1 ==> 2
61 1 0.154 2 --> 1
83 1 0.200 0 ==> 1
106 1 0.107 2 ==> 3
110 1 0.143 2 --> 1
127 2 0.138 3 --> 1
129 1 0.154 1 --> 2
167 1 0.154 0 --> 1
179 1 0.333 1 --> 2
184 1 0.143 0 --> 1
186 1 0.158 3 ==> 4
187 2 0.158 1 ==> 3
188 1 0.250 0 ==> 1
194 2 0.143 0 --> 2
195 1 0.182 2 --> 0
221 1 0.143 1 ==> 2
245 2 0.222 2 --> 0
247 1 0.200 0 --> 1
252 1 0.182 1 --> 0
255 1 0.200 0 --> 1
260 2 0.250 2 ==> 0
263 1 0.222 1 ==> 0
264 1 0.200 1 --> 0
node_78 --> node_77 2 1 0.167 0 --> 1
53 1 0.125 0 --> 1
54 1 0.200 1 --> 0
66 1 0.286 1 --> 0
71 1 0.111 1 --> 2

97 1 0.125 0 --> 1
105 1 0.133 1 --> 2
112 1 0.125 1 --> 0
143 1 0.200 0 --> 1
185 1 0.250 2 --> 1
220 2 0.111 0 ==> 2
269 1 0.222 1 --> 0
node_77 --> node_76 60 2 0.158 0 --> 2
194 1 0.143 2 --> 1
node_76 --> Lophostoma evotis 2 1 0.167 1 --> 0
5 1 0.273 2 ==> 0
14 1 0.667 0 ==> 1
25 1 0.200 2 ==> 3
82 1 0.500 0 ==> 1
84 1 0.200 0 ==> 3
100 1 0.333 0 ==> 1
107 1 0.333 1 ==> 0
108 1 0.333 0 ==> 2
111 1 0.111 1 ==> 0
123 1 0.167 1 ==> 2
124 1 0.333 0 ==> 2
129 1 0.154 2 --> 1
146 1 0.250 1 ==> 0
148 1 0.143 1 ==> 2
150 1 0.200 0 ==> 1
161 1 0.167 0 ==> 1
177 2 0.500 0 ==> 2
179 1 0.333 2 --> 1
195 1 0.182 0 ==> 1
214 3 0.333 3 ==> 0
233 1 0.286 1 ==> 0
235 1 0.250 1 ==> 2
259 1 0.125 0 ==> {12}
node_76 --> Phyllostomus hastatus hastatus 5 1 0.273 2 --> 1
33 1 0.500 1 ==> 0
34 1 0.158 0 ==> 1
47 2 0.200 2 ==> 0
53 1 0.125 1 --> 0
59 1 0.125 0 ==> 1
61 1 0.154 1 ==> 0
66 1 0.286 0 ==> 1
67 1 0.143 2 ==> 1
71 1 0.111 2 --> 1
78 1 0.200 0 --> 1
97 1 0.125 1 --> 0
100 1 0.333 0 ==> 2
105 1 0.133 2 --> 1
106 3 0.107 3 ==> 0
112 2 0.125 0 ==> 2
120 1 0.143 1 ==> 0
134 1 0.200 0 --> 1

160 1 0.111 0 ==> 1
167 1 0.154 1 ==> 2
174 1 0.167 0 ==> 1
190 1 0.167 0 ==> 1
194 1 0.143 1 ==> 0
210 1 0.200 2 ==> 1
220 1 0.111 2 ==> 1
221 1 0.143 2 ==> 1
253 1 0.278 1 ==> 0
277 1 0.091 1 ==> 0
node_76 --> node_75 1 1 0.100 1 --> 0
18 1 0.167 0 --> 1
20 1 0.333 0 --> 1
24 1 0.100 1 --> 0
29 2 0.214 1 --> 3
51 1 0.167 0 --> 1
58 1 0.167 2 --> 1
60 2 0.158 2 --> 0
120 1 0.143 1 --> 2
145 1 0.667 0 --> 2
151 1 0.400 0 --> 1
153 1 0.222 1 --> 0
154 1 0.143 0 --> 1
162 1 0.364 2 --> 1
165 1 0.125 1 --> 0
185 1 0.250 1 --> 0
194 1 0.143 1 --> 2
234 1 0.200 0 ==> 1
249 1 0.111 0 ==> 1
278 1 0.091 1 --> 0
node_75 --> Phylloderma stenops stenops 78 1 0.200 0 --> 1
123 1 0.167 1 ==> 2
129 2 0.154 2 ==> 0
226 1 0.111 0 ==> 1
node_75 --> node_74 115 1 0.333 1 --> 0
118 1 0.375 4 --> 1
122 1 0.250 3 --> 4
123 1 0.167 1 --> 0
127 1 0.138 1 --> 0
233 1 0.286 1 ==> 0
node_74 --> Notonycteris magdalenensis 243 1 0.333 0 ==> 1
263 1 0.222 0 ==> 1
node_77 --> Tonatia saurophila saurophila 190 1 0.167 0 ==> 1
195 1 0.182 0 ==> 1
202 1 0.182 1 ==> 0
node_78 --> Trachops cirrhosus cirrhosus 14 1 0.667 0 ==> 1
16 1 0.222 1 ==> 2
26 2 0.250 0 ==> 2
34 1 0.158 0 ==> 1
58 1 0.167 2 ==> 1
61 1 0.154 1 ==> 0

	84	1	0.200	0 --> 2
	85	1	0.200	1 ==> 0
	86	1	0.400	0 ==> 1
	87	1	0.250	0 ==> 1
	100	1	0.333	0 ==> 3
	103	1	0.200	1 ==> 2
	108	1	0.333	0 ==> 1
	111	1	0.111	1 ==> 0
	112	1	0.125	1 ==> 2
	120	1	0.143	1 ==> 2
	122	1	0.250	3 ==> 4
	124	1	0.333	0 ==> 1
	134	1	0.200	0 --> 1
	140	1	0.222	2 ==> 1
	152	1	0.333	0 ==> 1
	165	1	0.125	1 ==> 2
	177	1	0.500	0 ==> 1
	182	1	0.200	0 ==> 1
	192	1	0.214	3 ==> 1
	193	1	0.200	1 ==> 0
	203	1	0.250	1 ==> 0
	210	1	0.200	2 ==> 1
	223	1	0.222	1 ==> 2
	235	1	0.250	1 ==> 2
	236	1	0.143	0 ==> 2
	237	1	0.333	1 ==> 2
	267	1	0.154	1 ==> 0
	271	1	0.200	0 --> 1
	277	1	0.091	1 ==> 0
	278	1	0.091	1 ==> 0
node_84 --> node_83	13	1	0.273	3 --> 2
	31	1	0.286	0 --> 1
	49	1	0.286	1 --> 2
	55	1	0.167	0 --> 1
	60	1	0.158	0 --> 1
	65	1	0.200	1 ==> 0
	78	1	0.200	0 --> 1
	106	1	0.107	2 --> 1
	120	1	0.143	1 --> 0
	122	1	0.250	3 --> 2
	123	1	0.167	1 ==> 0
	128	1	0.143	1 --> 0
	173	1	0.286	0 --> 1
	175	1	0.143	2 --> 1
	196	1	0.200	1 --> 0
	219	1	0.125	0 ==> 1
	253	1	0.278	1 --> 0
	269	1	0.222	1 ==> 2
	274	1	0.250	2 ==> 1
	275	1	0.333	1 --> 2
node_83 --> node_81	5	1	0.273	2 --> 1

	21	1	0.125	0 ==> 1	
	25	2	0.200	2 --> 0	
	26	1	0.250	0 --> 1	
	29	1	0.214	2 ==> 3	
	34	1	0.158	0 ==> 1	
	37	1	0.200	1 ==> 0	
	50	1	0.400	1 ==> 0	
	63	1	0.125	0 ==> 1	
	84	1	0.200	0 --> 2	
	105	1	0.133	1 ==> 2	
	112	1	0.125	1 --> 0	
	117	1	0.167	1 --> 0	
	119	1	0.333	1 --> 0	
	129	1	0.154	1 --> 0	
	131	1	0.444	2 ==> 1	
	139	4	0.227	5 ==> 1	
	140	2	0.222	2 ==> 0	
	152	1	0.333	0 --> 1	
	159	1	0.250	0 ==> 1	
	160	1	0.111	0 ==> 1	
	172	1	0.167	0 ==> 1	
	178	1	0.250	0 ==> 1	
	180	1	0.500	0 ==> 1	
	182	1	0.200	0 ==> 1	
	192	1	0.214	3 ==> 0	
	206	1	0.286	2 ==> 1	
	220	1	0.111	0 --> 1	
	249	1	0.111	0 --> 1	
	253	1	0.278	0 --> 4	
	280	1	0.125	2 ==> 1	
node_81 --> node_80	1	1	0.100	1 --> 0	
	12	1	0.455	0 --> 1	
	13	1	0.273	2 --> 1	
	63	1	0.125	1 --> 2	
	71	1	0.111	1 --> 2	
	106	2	0.107	1 ==> 3	
	114	1	0.250	0 --> 1	
	115	1	0.333	1 --> 0	
	127	1	0.138	3 --> 2	
	145	1	0.667	0 --> 4	
	149	1	0.333	0 --> 1	
	151	1	0.400	0 --> 3	
	154	1	0.143	0 --> 1	
	158	1	0.200	0 --> 1	
	164	1	0.222	2 --> 1	
	165	1	0.125	1 ==> 0	
	170	1	0.333	0 --> 1	
	203	1	0.250	1 ==> 0	
	235	1	0.250	1 ==> 0	
	236	1	0.143	0 ==> 1	
	259	1	0.125	0 ==> 1	

node_80 --> Anoura geoffroyi peruana 40 3 0.231 3 ==> 0
56 1 0.250 0 ==> 1
70 1 0.400 2 ==> 1
82 1 0.500 0 ==> 1
84 1 0.200 2 ==> 3
85 1 0.200 1 ==> 0
87 1 0.250 0 ==> 1
111 1 0.111 1 ==> 0
120 1 0.143 0 --> 1
131 1 0.444 1 ==> 0
138 1 1.000 1 ==> 0
139 1 0.227 1 --> 2
144 1 1.000 0 ==> 1
150 1 0.200 0 ==> 1
173 1 0.286 1 ==> 2
176 1 0.400 0 ==> 1
186 1 0.158 3 ==> 2
193 1 0.200 1 ==> 0
202 1 0.182 1 ==> 2
206 1 0.286 1 ==> 0
210 2 0.200 2 ==> 0
219 1 0.125 1 ==> 0
244 1 0.333 0 ==> 1
253 1 0.278 4 ==> 3
263 1 0.222 1 ==> 0
267 1 0.154 1 ==> 0
278 1 0.091 1 ==> 0
node_80 --> node_79 23 1 0.133 0 ==> 1
25 2 0.200 0 --> 2
36 1 0.333 1 --> 0
58 1 0.167 2 ==> 1
97 1 0.125 0 --> 1
98 1 0.286 4 --> 1
128 1 0.143 0 --> 1
133 1 0.500 0 --> 1
139 1 0.227 1 --> 0
140 1 0.222 0 --> 1
142 1 0.500 0 --> 1
220 1 0.111 1 --> 0
243 1 0.333 0 ==> 1
249 1 0.111 1 --> 0
272 1 0.500 0 --> 1
274 1 0.250 1 --> 0
277 1 0.091 1 --> 0
node_79 --> Phyllonycteris aphylla 5 1 0.273 1 ==> 2
13 1 0.273 1 --> 2
29 1 0.214 3 ==> 2
31 1 0.286 1 ==> 0
34 1 0.158 1 ==> 0
37 1 0.200 0 ==> 1
47 1 0.200 1 ==> 2

53	1	0.125	0 ==> 1
54	1	0.200	1 ==> 0
58	1	0.167	1 ==> 0
63	2	0.125	2 ==> 0
65	1	0.200	0 ==> 1
66	1	0.286	1 ==> 0
68	1	0.500	0 ==> 1
75	1	0.333	0 ==> 1
76	1	0.500	0 ==> 1
81	2	0.500	1 ==> 3
93	1	0.200	0 ==> 1
102	1	0.200	2 ==> 1
110	1	0.143	2 ==> 1
127	2	0.138	2 ==> 0
153	1	0.222	1 ==> 2
164	1	0.222	1 --> 2
170	1	0.333	1 --> 0
172	1	0.167	1 ==> 0
174	1	0.167	0 ==> 1
192	1	0.214	0 ==> 3
202	1	0.182	1 ==> 0
204	1	0.500	1 ==> 2
207	1	0.100	0 ==> 1
211	1	0.500	0 ==> 1
217	1	0.286	2 ==> 1
221	1	0.143	1 ==> 0
223	1	0.222	1 ==> 0
233	1	0.286	1 ==> 2
234	1	0.200	0 ==> 1
237	1	0.333	1 ==> 0
238	1	0.500	0 ==> 1
243	1	0.333	1 ==> 2
252	1	0.182	1 ==> 2
253	1	0.278	4 --> 2
259	1	0.125	1 ==> 2
262	2	0.375	0 ==> 2
264	1	0.200	1 ==> 2
node_79 --> Leptonycteris yerbabuenae 1	1	0.100	0 --> 1
19	1	0.200	0 ==> 1
23	1	0.133	1 ==> 2
26	1	0.250	1 ==> 2
31	1	0.286	1 ==> 2
34	1	0.158	1 ==> 2
84	1	0.200	2 --> 0
98	1	0.286	1 --> 3
110	2	0.143	2 ==> 4
114	1	0.250	1 --> 0
118	1	0.375	4 ==> 2
121	1	0.500	0 ==> 1
122	2	0.250	2 ==> 0
127	2	0.138	2 ==> 4

130 1 0.333 0 ==> 1
163 1 0.200 0 ==> 1
166 1 1.000 0 ==> 1
204 1 0.500 1 ==> 0
206 1 0.286 1 ==> 2
222 1 0.500 0 ==> 1
250 1 0.222 1 ==> 2
265 1 0.500 0 ==> 1
node_81 --> Lonchophylla thomasi 17 1 0.200 0 ==> 1
24 1 0.100 1 --> 0
31 1 0.286 1 ==> 2
34 1 0.158 1 ==> 2
53 1 0.125 0 ==> 1
54 1 0.200 1 ==> 0
60 1 0.158 1 ==> 2
72 1 1.000 0 ==> 1
110 2 0.143 2 ==> 4
127 1 0.138 3 ==> 4
148 1 0.143 1 --> 2
183 1 1.000 1 ==> 0
187 1 0.158 1 --> 2
250 1 0.222 1 ==> 2
node_83 --> node_82 50 1 0.400 1 --> 2
51 1 0.167 0 ==> 1
59 1 0.125 0 ==> 1
69 1 0.154 1 ==> 0
81 2 0.500 1 ==> 3
86 1 0.400 0 ==> 1
93 1 0.200 0 --> 1
98 1 0.286 4 --> 0
102 1 0.200 2 --> 1
103 1 0.200 1 --> 0
112 1 0.125 1 --> 2
118 1 0.375 4 --> 0
132 1 0.250 0 --> 1
146 1 0.250 1 --> 0
162 1 0.364 2 --> 1
176 1 0.400 0 --> 1
186 1 0.158 3 --> 2
214 1 0.333 3 --> 2
217 1 0.286 2 ==> 1
227 1 0.286 0 ==> 1
240 1 0.250 2 --> 1
250 1 0.222 1 ==> 0
271 1 0.200 0 --> 1
277 1 0.091 1 --> 0
node_82 --> Carollia perspicillata 2 1 0.167 0 ==> 1
5 1 0.273 2 --> 0
13 1 0.273 2 --> 3
17 1 0.200 0 ==> 1
31 1 0.286 1 --> 0

38	1	0.250	0 ==> 1
56	1	0.250	0 ==> 1
57	2	0.286	0 ==> 2
60	1	0.158	1 --> 0
86	1	0.400	1 ==> 2
98	1	0.286	0 --> 1
105	1	0.133	1 ==> 0
110	2	0.143	2 ==> 4
118	1	0.375	0 --> 1
122	1	0.250	2 --> 3
127	1	0.138	3 ==> 4
142	1	0.500	0 ==> 1
148	1	0.143	1 --> 2
153	1	0.222	1 ==> 0
158	1	0.200	0 ==> 1
167	1	0.154	0 --> 1
169	1	0.200	0 ==> 1
202	1	0.182	1 ==> 0
207	1	0.100	0 ==> 1
210	1	0.200	2 ==> 1
243	1	0.333	0 ==> 1
251	1	0.333	0 ==> 1
253	1	0.278	0 --> 2
254	1	0.182	2 ==> 1
262	2	0.375	0 ==> 2
276	1	0.500	0 ==> 1
node_82 --> Artibeus jamaicensis 1	1	0.100	1 ==> 0
12	1	0.455	0 ==> 1
16	1	0.222	1 ==> 0
23	1	0.133	0 ==> 1
24	1	0.100	1 --> 0
32	1	1.000	1 ==> 2
47	1	0.200	1 ==> 2
49	1	0.286	2 --> 1
55	1	0.167	1 --> 0
58	1	0.167	2 ==> 3
60	1	0.158	1 ==> 2
64	1	0.667	1 ==> 2
66	1	0.286	1 ==> 2
67	1	0.143	2 ==> 1
75	1	0.333	0 ==> 1
78	1	0.200	1 ==> 2
101	1	0.500	0 ==> 1
104	1	1.000	0 ==> 1
106	1	0.107	1 ==> 0
107	1	0.333	1 ==> 0
110	2	0.143	2 ==> 0
114	1	0.250	0 ==> 1
121	1	0.500	0 ==> 1
122	2	0.250	2 ==> 0
126	1	0.500	1 ==> 0

	131	2	0.444	2 ==> 4
	133	1	0.500	0 ==> 1
	145	1	0.667	0 ==> 2
	151	1	0.400	0 ==> 1
	162	1	0.364	1 --> 3
	165	1	0.125	1 ==> 0
	174	1	0.167	0 ==> 1
	192	1	0.214	3 ==> 1
	193	1	0.200	1 ==> 0
	195	1	0.182	2 ==> 1
	221	1	0.143	1 ==> 0
	223	1	0.222	1 ==> 0
	233	1	0.286	1 ==> 2
	235	1	0.250	1 ==> 0
	237	1	0.333	1 ==> 0
	238	1	0.500	0 ==> 1
	244	1	0.333	0 ==> 1
	252	1	0.182	1 ==> 2
	255	1	0.200	0 --> 1
	259	1	0.125	0 ==> 1
	263	1	0.222	1 ==> 2
	264	1	0.200	1 ==> 2
	266	1	0.500	0 ==> 1
	267	1	0.154	1 ==> 2
	268	1	0.500	0 ==> 1
	271	1	0.200	1 ==> 2
	272	1	0.500	0 ==> 1
	274	1	0.250	1 ==> 0
node_70 --> node_49	1	1	0.100	1 --> 0
	5	1	0.273	1 --> 2
	16	1	0.222	0 --> 1
	24	1	0.100	0 --> 1
	74	1	0.200	0 --> 1
	116	1	0.167	0 ==> 1
	120	1	0.143	1 --> 2
	122	1	0.250	3 --> 2
	127	1	0.138	1 --> 0
	135	1	0.200	0 ==> 1
	160	1	0.111	0 ==> 1
	165	1	0.125	1 --> 2
	190	1	0.167	0 --> 1
	195	1	0.182	0 --> 1
	208	1	0.250	0 --> 1
	210	1	0.200	2 --> 1
	245	1	0.222	0 --> 1
	250	1	0.222	1 ==> 0
	275	1	0.333	1 --> 2
	278	1	0.091	1 --> 0
node_49 --> Mormoops blainvillei	11	1	0.250	1 ==> 0
	21	1	0.125	0 ==> 1
	23	1	0.133	1 ==> 2

	34	1	0.158	0 ==> 1
	47	1	0.200	1 ==> 0
	55	1	0.167	0 ==> 1
	58	1	0.167	2 ==> 3
	59	1	0.125	0 --> 1
	70	1	0.400	2 ==> 1
	100	1	0.333	0 ==> 3
	106	1	0.107	2 --> 1
	111	1	0.111	1 --> 0
	112	1	0.125	1 ==> 0
	124	1	0.333	0 ==> 1
	129	1	0.154	1 ==> 0
	158	1	0.200	0 --> 1
	173	1	0.286	0 --> 1
	182	1	0.200	0 ==> 1
	187	1	0.158	2 --> 1
	232	1	0.200	1 ==> 0
	247	1	0.200	1 ==> 0
	253	1	0.278	1 ==> 2
	269	1	0.222	0 ==> 1
	279	1	0.500	1 ==> 0
	280	1	0.125	2 ==> 1
node_49 --> node_48	18	1	0.167	0 --> 1
	26	1	0.250	0 ==> 1
	63	1	0.125	0 ==> 1
	67	1	0.143	2 --> 1
	96	1	0.333	0 ==> 1
	102	1	0.200	2 ==> 1
	103	1	0.200	1 ==> 0
	106	1	0.107	2 ==> 3
	112	1	0.125	1 ==> 2
	118	1	0.375	4 ==> 2
	129	1	0.154	1 ==> 2
	148	2	0.143	2 ==> 0
	153	1	0.222	1 ==> 0
	175	1	0.143	1 ==> 2
	179	1	0.333	1 ==> 2
	184	1	0.143	0 --> 1
	186	1	0.158	3 ==> 4
	187	1	0.158	2 ==> 3
	188	1	0.250	0 ==> 1
	194	1	0.143	2 ==> 1
	217	1	0.286	2 ==> 1
	221	1	0.143	1 ==> 0
	226	1	0.111	0 ==> 1
	236	1	0.143	0 --> 1
node_48 --> Pteronotus davyi	53	1	0.125	0 ==> 1
	67	1	0.143	1 ==> 0
	110	1	0.143	1 ==> 0
	139	1	0.227	1 ==> 0
	195	1	0.182	1 --> 0

208 1 0.250 1 --> 0
220 1 0.111 1 ==> 0
236 1 0.143 1 --> 2
254 1 0.182 2 ==> 1
270 1 0.333 0 ==> 1
278 1 0.091 0 --> 1
node_48 --> Pteronotus parnellii 16 1 0.222 1 --> 0
19 1 0.200 0 ==> 1
23 1 0.133 1 ==> 0
69 1 0.154 0 --> 1
77 1 0.200 0 ==> 1
97 1 0.125 1 --> 0
98 1 0.286 4 ==> 2
122 1 0.250 2 --> 3
127 1 0.138 0 --> 1
132 1 0.250 0 ==> 1
152 1 0.333 1 ==> 0
161 1 0.167 0 ==> 1
165 2 0.125 2 ==> 0
177 1 0.500 0 ==> 1
190 1 0.167 1 --> 0
192 1 0.214 3 ==> 1
194 1 0.143 1 ==> 0
206 1 0.286 2 ==> 1
210 1 0.200 1 --> 2
215 1 0.143 0 ==> 1
245 1 0.222 1 --> 0
259 1 0.125 0 ==> 1
260 1 0.250 1 ==> 0
267 1 0.154 0 --> 1
271 1 0.200 1 ==> 0
274 1 0.250 2 ==> 1
node_69 --> node_54 35 1 0.500 0 --> 1
58 1 0.167 2 --> 3
83 1 0.200 0 ==> 1
84 1 0.200 0 --> 2
96 1 0.333 0 --> 1
100 1 0.333 0 --> 3
103 1 0.200 1 ==> 0
129 1 0.154 1 ==> 2
155 1 0.200 1 --> 0
163 1 0.200 0 --> 1
165 1 0.125 1 --> 0
172 1 0.167 0 ==> 1
186 1 0.158 3 ==> 2
192 1 0.214 3 --> 2
224 1 1.000 0 --> 1
226 1 0.111 0 --> 1
259 1 0.125 0 --> 1
node_54 --> node_52 23 1 0.133 1 --> 2
29 1 0.214 1 --> 0

	105	1	0.133	1 --> 0
	112	1	0.125	1 --> 2
	120	1	0.143	1 --> 0
	122	1	0.250	3 --> 2
	123	1	0.167	1 --> 0
	175	1	0.143	1 --> 2
	184	1	0.143	0 --> 1
	196	1	0.200	1 --> 0
	205	1	1.000	0 --> 1
	221	1	0.143	1 --> 0
	245	1	0.222	0 ==> 1
	253	1	0.278	1 --> 0
	267	1	0.154	0 --> 1
	269	1	0.222	0 ==> 1
	277	1	0.091	1 --> 0
node_52 --> node_50	13	1	0.273	2 ==> 3
	24	1	0.100	0 --> 1
	46	1	0.250	0 ==> 1
	67	1	0.143	1 ==> 0
	78	1	0.200	1 ==> 0
	85	1	0.200	1 ==> 0
	96	1	0.333	1 --> 0
	100	1	0.333	3 --> 0
	108	1	0.333	0 ==> 1
	110	1	0.143	2 ==> 1
	124	1	0.333	0 ==> 1
	127	1	0.138	2 ==> 1
	139	1	0.227	1 ==> 2
	145	1	0.667	0 ==> 1
	148	1	0.143	2 ==> 1
	150	1	0.200	0 ==> 1
	155	1	0.200	0 --> 1
	158	1	0.200	1 ==> 0
	160	1	0.111	0 ==> 1
	161	1	0.167	0 ==> 1
	163	1	0.200	1 --> 0
	174	1	0.167	0 ==> 1
	189	1	1.000	1 ==> 0
	192	1	0.214	2 --> 1
	193	1	0.200	1 ==> 0
	195	1	0.182	0 ==> 1
	198	1	0.333	0 ==> 1
	202	1	0.182	1 --> 0
	207	1	0.100	0 --> 1
	217	1	0.286	2 ==> 1
	220	1	0.111	1 --> 0
	222	1	0.500	0 ==> 1
	223	1	0.222	1 ==> 2
	226	1	0.111	1 --> 0
	231	1	0.333	0 ==> 1
	242	1	0.333	2 --> 1

	250	1	0.222	1 ==> 0
	261	1	1.000	0 ==> 1
	271	1	0.200	1 ==> 0
node_50 --> Noctilio albiventris	62	1	0.667	0 ==> 1
	90	1	0.500	1 ==> 0
	107	1	0.333	1 ==> 0
	111	1	0.111	0 ==> 1
	122	1	0.250	2 --> 3
	126	1	0.500	1 ==> 0
	128	1	0.143	0 ==> 1
	197	1	0.333	0 ==> 1
	219	1	0.125	0 ==> 1
	236	1	0.143	0 ==> 2
	247	1	0.200	1 ==> 0
	259	1	0.125	1 ==> 2
node_50 --> Noctilio leporinus	109	1	0.500	0 ==> 1
	110	1	0.143	1 ==> 0
	125	1	0.500	0 ==> 1
node_52 --> node_51	1	1	0.100	1 --> 0
	2	1	0.167	0 ==> 1
	9	1	0.250	1 --> 0
	13	1	0.273	2 ==> 1
	21	1	0.125	0 ==> 1
	58	1	0.167	3 --> 2
	59	1	0.125	1 ==> 0
	61	1	0.154	1 ==> 0
	65	1	0.200	1 ==> 0
	67	1	0.143	1 --> 2
	92	1	0.250	0 ==> 1
	110	2	0.143	2 ==> 4
	116	1	0.167	0 ==> 1
	127	2	0.138	2 ==> 4
	158	1	0.200	1 ==> 2
	162	1	0.364	2 ==> 0
	164	1	0.222	2 ==> 1
	215	1	0.143	1 ==> 0
	240	1	0.250	2 ==> 1
	264	1	0.200	0 ==> 1
	274	1	0.250	2 ==> 1
	278	1	0.091	1 --> 0
node_51 --> Furipterus horrens	34	3	0.158	0 ==> 3
	55	1	0.167	0 ==> 1
	60	1	0.158	1 --> 2
	63	2	0.125	0 ==> 2
	69	1	0.154	1 ==> 2
	74	1	0.200	0 --> 1
	77	1	0.200	0 ==> 1
	100	1	0.333	3 --> 1
	105	1	0.133	0 --> 1
	106	1	0.107	1 ==> 0
	123	1	0.167	0 --> 1

131 1 0.444 1 ==> 0
135 1 0.200 0 ==> 1
143 1 0.200 0 ==> 1
172 1 0.167 1 ==> 0
192 1 0.214 2 --> 0
196 1 0.200 0 --> 1
227 1 0.286 0 ==> 1
232 1 0.200 1 ==> 0
236 1 0.143 0 ==> 1
252 1 0.182 0 ==> 1
267 1 0.154 1 --> 0
277 1 0.091 0 --> 1
280 1 0.125 2 ==> 1
node_51 --> Amorphochilus schnablii 23 2 0.133 2 ==> 0
69 1 0.154 1 ==> 0
83 1 0.200 1 ==> 0
103 1 0.200 0 ==> 1
120 1 0.143 0 --> 1
156 1 0.500 2 --> 1
186 1 0.158 2 ==> 1
187 1 0.158 2 --> 1
253 1 0.278 0 --> 1
259 1 0.125 1 --> 0
279 1 0.500 1 ==> 0
node_54 --> node_53 25 1 0.200 2 ==> 3
40 3 0.231 3 ==> 0
51 1 0.167 0 ==> 1
60 1 0.158 1 --> 2
63 1 0.125 0 ==> 1
74 1 0.200 0 --> 1
91 1 1.000 1 ==> 0
105 1 0.133 1 ==> 2
108 1 0.333 0 ==> 2
120 1 0.143 1 ==> 2
124 1 0.333 0 ==> 2
154 1 0.143 0 ==> 1
162 1 0.364 2 ==> 1
210 1 0.200 2 ==> 1
221 1 0.143 1 ==> 2
232 1 0.200 1 ==> 0
249 1 0.111 0 ==> 1
252 1 0.182 0 ==> 1
260 1 0.250 1 --> 0
275 1 0.333 1 --> 0
node_53 --> Thyroptera discifera 56 1 0.250 0 ==> 1
57 1 0.286 0 ==> 1
67 1 0.143 1 --> {02}
100 1 0.333 3 --> 2
106 2 0.107 1 ==> 3
110 1 0.143 2 ==> 3
116 1 0.167 0 ==> 1

	123	1	0.167	1 ==> 2
	127	1	0.138	2 ==> 3
	165	1	0.125	0 --> 1
	178	1	0.250	0 ==> 1
	180	1	0.500	0 ==> 1
	186	1	0.158	2 ==> 1
	190	1	0.167	0 ==> 1
	202	1	0.182	1 --> 0
	204	1	0.500	1 ==> 2
	214	3	0.333	3 ==> 0
	220	1	0.111	1 ==> 2
	250	1	0.222	1 ==> 2
	259	1	0.125	1 --> 0
node_53 --> Thyroptera tricolor	8	1	0.500	0 ==> 1
	21	1	0.125	0 ==> 1
	33	1	0.500	1 ==> 0
	69	1	0.154	1 ==> 0
	106	1	0.107	1 ==> 0
	117	1	0.167	1 ==> 0
	139	1	0.227	1 ==> 0
	160	1	0.111	0 ==> 1
	192	1	0.214	2 --> 3
	207	1	0.100	0 --> 1
	220	1	0.111	1 --> 0
	233	1	0.286	1 ==> 0
	236	1	0.143	0 ==> 2
	240	1	0.250	2 ==> 1
	254	2	0.182	2 ==> 0
	255	1	0.200	1 ==> 0
	262	1	0.375	0 ==> 1
	280	1	0.125	2 ==> 1
node_68 --> node_58	9	1	0.250	1 --> 0
	15	1	0.250	0 --> 1
	25	1	0.200	2 --> 3
	51	1	0.167	0 --> 1
	53	1	0.125	0 --> 1
	57	1	0.286	0 --> 1
	71	1	0.111	2 ==> 1
	92	1	0.250	0 ==> 1
	109	1	0.500	0 --> 1
	112	1	0.125	1 --> 0
	120	1	0.143	1 --> 0
	125	1	0.500	0 --> 1
	129	1	0.154	1 --> 0
	148	2	0.143	2 --> 0
	150	1	0.200	0 ==> 1
	161	1	0.167	0 ==> 1
	169	1	0.200	0 ==> 1
	175	1	0.143	1 ==> 2
	187	2	0.158	2 --> 0
	197	1	0.333	0 --> 1

	198	1	0.333	0 --> 1
	207	1	0.100	0 --> 1
	242	1	0.333	2 --> 1
	252	1	0.182	0 ==> 1
	253	1	0.278	1 --> 0
	271	1	0.200	1 ==> 0
node_58 --> node_56	97	1	0.125	1 --> 0
	227	1	0.286	0 ==> 1
	253	1	0.278	0 --> 2
	260	1	0.250	1 --> 0
node_56 --> node_55	69	1	0.154	1 ==> 0
	98	1	0.286	4 ==> 3
	103	1	0.200	1 ==> 0
	117	1	0.167	1 --> 0
	236	1	0.143	0 --> 1
	249	1	0.111	0 --> 1
	259	1	0.125	0 --> 1
	264	1	0.200	0 ==> 1
	267	1	0.154	0 --> 1
	274	1	0.250	2 ==> 1
node_55 --> Vulcanops worthyae	77	1	0.200	0 ==> 1
	94	1	0.250	1 ==> 0
	103	1	0.200	0 ==> 1
	108	1	0.333	0 ==> 3
	110	1	0.143	2 ==> 1
	112	2	0.125	0 ==> 2
	115	1	0.333	1 ==> 0
	124	1	0.333	0 ==> 3
	127	1	0.138	2 ==> 1
	129	2	0.154	0 ==> 2
	134	1	0.200	1 ==> 0
	139	1	0.227	1 ==> 0
	143	1	0.200	0 ==> 1
	213	1	1.000	1 ==> 0
	214	1	0.333	3 ==> 2
	219	1	0.125	0 ==> 1
	220	1	0.111	1 --> 0
	221	1	0.143	1 ==> 2
	223	1	0.222	1 ==> 0
	226	1	0.111	0 ==> 1
	236	1	0.143	1 --> 0
	242	1	0.333	1 --> 2
	245	1	0.222	0 ==> 1
	254	1	0.182	2 ==> 1
	259	1	0.125	1 --> 0
node_55 --> Mystacina robusta	34	1	0.158	1 ==> 2
	56	1	0.250	0 ==> 1
	71	1	0.111	1 ==> 2
	98	1	0.286	3 ==> 4
	194	1	0.143	2 ==> 1
	249	1	0.111	1 --> 0

	253	1	0.278	2 ==> 1
node_55 --> Mystacina tuberculata	23	1	0.133	1 ==> 0
	49	1	0.286	0 ==> 1
	61	1	0.154	0 --> 1
	71	1	0.111	1 ==> 2
	94	1	0.250	1 ==> 0
	127	1	0.138	2 ==> 3
	165	1	0.125	1 ==> 2
	167	1	0.154	2 ==> 1
	200	1	0.500	1 --> 0
	210	1	0.200	2 ==> 1
	235	1	0.250	1 ==> 0
node_55 --> Mystacina miocenalis	74	1	0.200	0 ==> 1
	90	1	0.500	1 ==> 0
	122	1	0.250	3 ==> 2
	127	1	0.138	2 ==> 3
node_56 --> Icarops paradox	63	1	0.125	0 ==> 1
	67	1	0.143	1 ==> 0
	78	1	0.200	1 ==> 2
	116	1	0.167	0 ==> 1
	241	1	0.250	0 ==> 1
	277	1	0.091	1 ==> 0
	280	1	0.125	2 ==> 1
node_58 --> node_57	110	1	0.143	2 --> 1
	219	1	0.125	0 ==> 1
	220	1	0.111	1 --> 0
node_57 --> Icarops aenae	269	1	0.222	0 ==> 1
	280	1	0.125	2 ==> 1
node_67 --> node_66	7	1	1.000	0 --> 1
	58	1	0.167	2 --> 3
	110	1	0.143	2 --> 3
	122	1	0.250	3 --> 2
	127	1	0.138	2 --> 3
	141	1	0.500	0 --> 1
	162	1	0.364	2 --> 3
	240	1	0.250	2 ==> 1
node_66 --> Natalus stramineus	5	1	0.273	0 ==> 2
	13	1	0.273	2 --> 1
	21	1	0.125	0 --> 1
	23	1	0.133	1 ==> 0
	25	1	0.200	2 ==> 1
	34	1	0.158	1 ==> 0
	40	3	0.231	3 ==> 0
	47	1	0.200	1 ==> 0
	56	1	0.250	0 ==> 1
	60	2	0.158	2 ==> 0
	62	2	0.667	0 ==> 2
	65	1	0.200	1 ==> 0
	67	1	0.143	1 --> 2
	69	1	0.154	1 ==> 0
	85	1	0.200	1 ==> 0

	92	1	0.250	0 ==> 1
	98	1	0.286	4 ==> 3
	105	1	0.133	1 ==> 2
	108	1	0.333	0 ==> 2
	112	1	0.125	1 --> 0
	116	1	0.167	0 ==> 1
	120	1	0.143	1 --> 0
	127	1	0.138	3 ==> 4
	131	1	0.444	1 ==> 0
	135	1	0.200	0 ==> 1
	139	1	0.227	1 ==> 0
	160	1	0.111	0 ==> 1
	165	1	0.125	1 ==> 0
	184	1	0.143	1 ==> 0
	186	2	0.158	3 ==> 1
	187	2	0.158	2 --> 0
	192	1	0.214	3 ==> 2
	207	1	0.100	0 --> 1
	236	1	0.143	0 ==> 2
	245	1	0.222	0 ==> 1
	252	1	0.182	0 ==> 1
	259	2	0.125	0 ==> 2
	264	1	0.200	0 ==> 1
	273	1	1.000	0 ==> 1
	280	1	0.125	2 ==> 1
node_66 --> node_65	2	1	0.167	0 ==> 1
	13	1	0.273	2 --> 3
	16	1	0.222	1 --> 2
	47	1	0.200	1 ==> 2
	51	1	0.167	0 --> 1
	67	1	0.143	1 ==> 0
	71	1	0.111	2 ==> 1
	77	1	0.200	0 ==> 1
	78	1	0.200	1 --> 0
	97	1	0.125	1 --> 0
	136	1	0.500	1 ==> 0
	154	1	0.143	0 ==> 1
	158	1	0.200	1 ==> 0
	163	1	0.200	0 ==> 1
	164	1	0.222	1 ==> 2
	167	1	0.154	2 --> 1
	169	1	0.200	0 ==> 1
	176	2	0.400	0 --> 2
	182	1	0.200	0 --> 1
	242	1	0.333	2 --> 1
	254	2	0.182	2 ==> 0
	267	1	0.154	0 --> 1
	271	1	0.200	1 ==> 0
	275	1	0.333	2 --> 1
node_65 --> Chalinolobus gouldii	12	2	0.455	1 ==> {34}
	24	1	0.100	1 ==> 0

48 2 1.000 0 ==> 2
100 1 0.333 0 ==> 3
120 1 0.143 1 ==> 2
122 1 0.250 2 --> 3
140 1 0.222 0 ==> 1
162 1 0.364 3 --> 2
175 1 0.143 1 ==> 2
186 1 0.158 3 ==> 4
187 1 0.158 2 ==> 3
188 1 0.250 0 ==> 1
192 1 0.214 3 --> 1
202 1 0.182 0 ==> 1
210 1 0.200 2 ==> 3
219 1 0.125 0 ==> 1
227 1 0.286 0 ==> 1
270 1 0.333 0 ==> 1
node_65 --> Chaerephon jobensis 15 1 0.250 0 ==> 1
26 1 0.250 0 ==> 1
29 1 0.214 0 --> 1
37 1 0.200 1 ==> 0
58 1 0.167 3 --> 2
61 1 0.154 0 --> 1
84 1 0.200 2 ==> 0
103 1 0.200 1 ==> 0
105 1 0.133 1 ==> 0
106 2 0.107 0 ==> 2
108 1 0.333 0 ==> 1
110 2 0.143 3 ==> 1
111 1 0.111 0 ==> 1
123 1 0.167 1 ==> 0
124 1 0.333 0 ==> 1
127 2 0.138 3 ==> 1
128 1 0.143 0 ==> 1
139 1 0.227 1 ==> 2
146 1 0.250 1 ==> 0
152 1 0.333 1 ==> 0
155 1 0.200 0 --> 1
185 1 0.250 0 ==> 1
190 1 0.167 0 ==> 1
195 1 0.182 0 ==> 1
197 1 0.333 0 ==> 1
198 1 0.333 0 ==> 1
215 1 0.143 1 ==> 0
221 1 0.143 1 ==> 0
222 1 0.500 0 ==> 2
226 1 0.111 0 ==> 1
229 1 1.000 0 ==> 1
231 1 0.333 0 ==> 1
249 1 0.111 0 ==> 1
253 1 0.278 1 ==> 0
277 1 0.091 0 --> 1

	278	1	0.091	0 --> 1
node_64 --> node_62	1	1	0.100	0 --> 1
	2	1	0.167	0 --> 1
	8	1	0.500	0 --> 1
	13	1	0.273	1 --> 0
	21	1	0.125	0 --> 1
	39	1	0.333	0 --> 1
	44	2	1.000	0 --> 2
	47	1	0.200	1 --> 2
	67	1	0.143	1 --> 2
	77	1	0.200	0 ==> 1
	79	1	1.000	0 ==> 1
	94	1	0.250	1 --> 0
	111	1	0.111	0 --> 1
	119	1	0.333	0 --> 1
	128	1	0.143	0 --> 1
	129	1	0.154	1 --> 2
	135	1	0.200	0 --> 1
	173	2	0.286	2 --> 0
	188	1	0.250	0 --> 1
	221	1	0.143	1 --> 2
	234	1	0.200	1 --> 0
node_62 --> node_61	40	2	0.231	2 --> 0
	60	2	0.158	2 --> 0
	65	1	0.200	1 --> 0
	69	1	0.154	1 --> 2
	78	1	0.200	1 --> 0
	86	1	0.400	0 --> 1
	98	1	0.286	0 --> 2
	102	1	0.200	2 --> 1
	105	1	0.133	1 --> 0
	110	2	0.143	2 --> 4
	118	1	0.375	4 --> 2
	123	1	0.167	1 --> 0
	127	2	0.138	2 --> 4
	132	1	0.250	0 --> 1
	134	1	0.200	1 --> 0
	139	1	0.227	1 --> 0
	146	1	0.250	1 --> 0
	148	1	0.143	2 --> 1
	153	1	0.222	1 --> 2
	159	1	0.250	0 --> 1
	160	1	0.111	0 --> 1
	162	1	0.364	2 --> 3
	175	1	0.143	1 ==> 2
	186	1	0.158	3 ==> 4
	187	1	0.158	2 ==> 3
	209	1	1.000	1 ==> 2
	219	1	0.125	0 --> 1
	227	1	0.286	0 ==> 1
	241	1	0.250	0 --> 1

249 1 0.111 0 ==> 1
252 1 0.182 0 ==> 1
264 1 0.200 0 ==> 1
267 1 0.154 0 --> 1
271 1 0.200 1 ==> 0
274 1 0.250 2 --> 1
node_61 --> node_60 194 1 0.143 2 --> 1
215 1 0.143 0 --> 1
254 1 0.182 2 --> 1
278 1 0.091 1 ==> 0
node_60 --> node_59 219 1 0.125 1 --> 0
228 1 1.000 0 ==> 1
277 1 0.091 0 --> 1
280 1 0.125 2 ==> 1
node_59 --> Myzopoda aurita 29 1 0.214 0 --> 1
92 1 0.250 0 ==> 1
152 1 0.333 1 ==> 0
172 1 0.167 0 ==> 1
241 1 0.250 1 --> 0
254 1 0.182 1 --> 2
259 1 0.125 0 ==> 1
267 1 0.154 1 ==> 0
node_59 --> Myzopoda schliemanni 34 1 0.158 1 ==> 0
43 1 0.600 2 ==> 3
94 1 0.250 0 --> 1
154 1 0.143 0 ==> 1
194 1 0.143 1 ==> 0
231 1 0.333 0 ==> 1
node_61 --> Phasmatonycteris phiomensis 253 1 0.278 1 ==> 2
node_62 --> Saccopteryx bilineata 3 1 1.000 0 ==> 1
17 1 0.200 0 --> 1
18 1 0.167 1 --> 0
23 1 0.133 1 ==> 2
24 1 0.100 1 ==> 0
34 1 0.158 1 ==> 2
43 2 0.600 2 ==> 0
46 1 0.250 0 ==> 1
58 1 0.167 2 ==> 1
59 1 0.125 1 ==> 0
60 1 0.158 2 ==> 3
61 1 0.154 0 --> 1
63 2 0.125 0 ==> 2
66 1 0.286 0 --> 1
69 1 0.154 1 ==> 0
85 1 0.200 1 ==> 0
95 1 1.000 0 ==> 1
103 1 0.200 1 ==> 0
105 1 0.133 1 ==> 2
106 3 0.107 0 ==> 3
108 1 0.333 0 ==> 2
110 1 0.143 2 ==> 1

116	1	0.167	0 ==> 1
117	1	0.167	0 ==> 1
122	1	0.250	3 ==> 2
123	1	0.167	1 ==> 2
124	1	0.333	0 ==> 1
139	1	0.227	1 --> 2
163	1	0.200	0 ==> 1
164	1	0.222	1 ==> 2
165	1	0.125	1 ==> 2
167	1	0.154	2 ==> 1
169	1	0.200	0 ==> 1
184	1	0.143	1 ==> 0
193	1	0.200	1 ==> 0
206	1	0.286	2 --> 1
208	1	0.250	0 ==> 1
223	1	0.222	1 ==> 2
226	1	0.111	0 ==> 1
232	1	0.200	1 ==> 0
236	1	0.143	0 ==> 1
247	1	0.200	1 ==> 0
250	1	0.222	1 ==> 0
255	1	0.200	1 ==> 0
259	1	0.125	0 ==> 1
277	1	0.091	0 --> 1
node_63 --> Hipposideros commersoni 1	1	0.100	0 --> 1
15	1	0.250	0 ==> 1
21	1	0.125	0 --> 1
23	1	0.133	1 ==> 2
25	1	0.200	2 ==> 3
34	1	0.158	1 ==> 2
35	1	0.500	0 --> 1
36	1	0.333	0 --> 1
39	1	0.333	0 --> 1
40	1	0.231	2 ==> 1
43	2	0.600	2 ==> 0
46	1	0.250	0 ==> 1
58	1	0.167	2 ==> 3
63	1	0.125	0 ==> 1
66	1	0.286	0 --> 1
83	1	0.200	0 --> 1
86	1	0.400	0 --> 1
98	1	0.286	0 --> 1
106	1	0.107	0 --> 1
131	1	0.444	1 --> 2
139	4	0.227	1 --> 5
140	2	0.222	0 --> 2
141	1	0.500	0 --> 1
143	1	0.200	0 --> 1
162	1	0.364	2 --> 3
174	1	0.167	0 ==> 1
202	1	0.182	0 --> 1

	207	1	0.100	0 --> 1
	220	2	0.111	0 --> 2
	251	2	0.333	0 --> 2
	263	2	0.222	0 --> 2
	276	1	0.500	0 --> 1
node_63 --> Pteropus scapulatus	12	1	0.455	1 ==> 0
	17	1	0.200	0 --> 1
	25	2	0.200	2 ==> 0
	29	1	0.214	2 ==> 3
	34	1	0.158	1 ==> 0
	37	1	0.200	1 ==> 0
	49	2	0.286	0 --> 2
	56	2	0.250	0 --> 2
	57	2	0.286	0 --> 2
	58	2	0.167	2 ==> 0
	59	1	0.125	1 ==> 0
	61	1	0.154	1 ==> 2
	67	1	0.143	1 --> 2
	68	1	0.500	0 ==> 1
	70	1	0.400	2 ==> 1
	73	1	1.000	0 ==> 1
	130	1	0.333	0 ==> 1
	145	1	0.667	0 ==> 4
	148	1	0.143	2 ==> 1
	149	1	0.333	0 ==> 1
	151	1	0.400	0 --> 3
	159	1	0.250	0 ==> 1
	160	1	0.111	0 ==> 1
	165	1	0.125	1 ==> 0
	167	2	0.154	2 ==> 0
	171	1	1.000	0 ==> 1
	178	1	0.250	0 --> 1
	185	2	0.250	0 --> 2
	194	1	0.143	1 ==> 0
	195	1	0.182	0 ==> 2
	210	1	0.200	1 ==> 0
	211	1	0.500	0 ==> 1
	235	1	0.250	1 ==> 0
	258	1	1.000	0 ==> 1
	266	1	0.500	0 ==> 1
	267	1	0.154	1 ==> 2
	268	1	0.500	0 ==> 1
	269	2	0.222	0 --> 2

****

2. Bayesian analysis of total evidence data - synapomorphies under Delayed Transformation (DELTRAN)

paup> set opt=deltran;

```
paup> describe /apolist;

Tree description:

  Unrooted tree(s) rooted using outgroup method
  Optimality criterion = parsimony
    Character-status summary:
      Of 292 total characters:
        112 characters are of type 'ord' (Wagner)
        180 characters are of type 'unord'
        All characters have equal weight
        37 characters are constant
        22 variable characters are parsimony-uninformative
        Number of parsimony-informative characters = 233
    Gaps are treated as "missing"
    Multistate taxa interpreted as uncertainty
    Character-state optimization: Delayed transformation (DELTRAN)

Tree 1 ("con 50 majrule") (rooted using user-specified outgroup)

Tree length = 1689
Consistency index (CI) = 0.2404
Homoplasy index (HI) = 0.7596
CI excluding uninformative characters = 0.2294
HI excluding uninformative characters = 0.7706
Retention index (RI) = 0.4491
Rescaled consistency index (RC) = 0.1080

                                /----- Speonycteris aurantiadens
                            /---88
                            |   \----- Micronycteris hirsuta
            /---------------------------87
            |               \---------- Mimon bennettii
            |
            |                   /----- Macrotus californicus
            +-------------------------------71
            |                   \----- Macrotus waterhousii
            |
            |                   /----- Desmodus rotundus
            |               /---72
        /---86              |   \----- Diaemus youngi
        |   |   /----------------------73
        |   |   |           \---------- Diphylla ecaudata
        |   |   |
        |   |   |         /--------------- Lophostoma evotis
        |   |   |         |
        |   |   |         +--------------- Phyllostomus hastatus hastatus
        |   |   |       /--76
        |   |   |       |   |   /---------- Phylloderma stenops stenops
        |   |   |       |   |   |
        |   \---85    /---77  \---75   /----- Notonycteris magdalenensis
```

```
          |       |       |   |      \---74
          |       |       |   |         \----- Notonycteris sucharadeus
          |       |   /---78   |
          |       |   |   |   \----------------- Tonatia saurophila saurophila
          |       |   |   |
       /---70       |   |   \---------------------- Trachops cirrhosus cirrhosus
       |   |       |   |
       |   |       |   |             /---------- Anoura geoffroyi peruana
       |   |       \---84             |
       |   |          |          /---80   /----- Phyllonycteris aphylla
       |   |          |          |   \---79
       |   |          |       /--81       \----- Leptonycteris yerbabuenae
       |   |          |       |   |
       |   |          \--------83   \--------------- Lonchophylla thomasi
       |   |                  |
       |   |                  |          /----- Carollia perspicillata
       |   |                  \------------82
       |   |                             \----- Artibeus jamaicensis
    /--69   |
    |   |   |                         /---------- Mormoops blainvillei
    |   |   |                         |
    |   |   \-------------------------------49   /----- Pteronotus davyi
    |   |                            \---48
    |   |                               \----- Pteronotus parnellii
    |   |
    |   |                               /----- Noctilio albiventris
    |   |                            /---50
    |   |                            |   \----- Noctilio leporinus
    |   |                        /---52
    |   |                        |   |   /----- Furipterus horrens
    |   |                        |   \---51
    |   \------------------------------54       \----- Amorphochilus schnablii
  /---68                           |
  |   |                          |       /----- Thyroptera discifera
  |   |                          \--------53
  |   |                               \----- Thyroptera tricolor
  |   |
  |   |                               /----- Vulcanops worthyae
  |   |                               |
  |   |                               +----- Mystacina robusta
  |   |                            /---55
  |   |                            |   +----- Mystacina tuberculata
/---67   |                            |   |
|   |   |                        /---56   \----- Mystacina miocenalis
|   |   |                        |   |
|   |   \----------------------------------58   \---------- Icarops paradox
|   |                            |
|   |                            |       /----- Icarops aenae
|   |                            \--------57
|   |                               \----- Icarops breviceps
|   |
```

```
    |    |                                        /---------- Natalus stramineus
    |    |                                        |
/---64   \------------------------------------------66    /----- Chalinolobus gouldii
|    |                                        \---65
|    |                                            \----- Chaerephon jobensis
|    |
|    |                                            /----- Myzopoda aurita
|    |                                        /---59
|    |                                        |    \----- Myzopoda schliemanni
|    |                                    /---60
63   |                                  /--61    \---------- Phasmatonycteris butleri
|    |                                  |    |
|    \---------------------------------------62   \-------------- Phasmatonycteris phiomensis
|                                        |
|                                        \------------------ Saccopteryx bilineata
|
+----------------------------------------------------------------- Hipposideros commersoni
|
\----------------------------------------------------------------- Pteropus scapulatus
```

Apomorphy lists:

```
              Branch              Character  Steps     CI   Change
-------------------------------------------------------------------
        node_63 --> node_64         5           1   0.273  1 ==> 0
                          29           1   0.214  2 ==> 1
                          50           2   0.400  0 --> 2
                          129          1   0.154  0 --> 1
                          136          1   0.500  0 --> 1
                          154          1   0.143  1 ==> 0
                          158          1   0.200  0 ==> 1
                          186          2   0.158  1 --> 3
                          194          1   0.143  1 ==> 2
                          210          1   0.200  1 ==> 2
                          214          1   0.333  2 --> 3
                          233          1   0.286  0 --> 1
                          242          1   0.333  0 --> 1
        node_64 --> node_67         11          1   0.250  0 ==> 1
                          52           1   1.000  0 --> 1
                          55           1   0.167  1 ==> 0
                          98           1   0.286  0 --> 4
                          102          1   0.200  1 --> 2
                          105          1   0.133  0 --> 1
                          123          1   0.167  0 --> 1
                          153          1   0.222  2 --> 1
                          206          1   0.286  1 --> 2
                          215          1   0.143  0 --> 1
                          274          1   0.250  1 --> 2
        node_67 --> node_68         12          1   0.455  1 ==> 0
                          40           1   0.231  2 --> 3
                          192          1   0.214  1 --> 3
```

	277	1	0.091	0 --> 1
node_68 --> node_69	13	1	0.273	1 --> 2
	34	1	0.158	1 ==> 0
	36	1	0.333	0 --> 1
	106	1	0.107	0 --> 1
	112	1	0.125	0 --> 1
	117	1	0.167	0 --> 1
	164	1	0.222	1 ==> 2
	187	1	0.158	0 --> 1
node_69 --> node_70	6	2	1.000	0 --> 2
	13	1	0.273	2 ==> 3
	29	1	0.214	1 ==> 2
	60	2	0.158	2 ==> 0
	110	1	0.143	2 ==> 1
	120	1	0.143	0 --> 1
	127	1	0.138	2 ==> 1
	128	1	0.143	0 ==> 1
	173	1	0.286	2 ==> 1
	196	1	0.200	0 --> 1
	202	1	0.182	0 --> 1
	215	1	0.143	1 ==> 0
	220	1	0.111	0 --> 1
	234	1	0.200	1 ==> 0
	241	1	0.250	0 ==> 1
	260	1	0.250	0 --> 1
node_70 --> node_86	1	1	0.100	0 --> 1
	5	1	0.273	0 --> 1
	49	1	0.286	0 ==> 1
	54	1	0.200	0 ==> 1
	67	1	0.143	1 --> 2
	74	1	0.200	1 --> 0
	111	1	0.111	0 --> 1
	131	1	0.444	1 ==> 2
	139	4	0.227	1 ==> 5
	140	2	0.222	0 ==> 2
	152	1	0.333	1 ==> 0
	158	1	0.200	1 --> 0
	173	1	0.286	1 --> 0
	263	1	0.222	0 ==> 1
node_86 --> node_87	19	1	0.200	0 --> 1
	25	1	0.200	2 ==> 3
	51	1	0.167	0 ==> 1
	69	1	0.154	1 --> 0
	71	1	0.111	2 ==> 1
	78	1	0.200	1 --> 0
	83	1	0.200	0 ==> 1
	108	1	0.333	0 ==> 1
	124	1	0.333	0 ==> 1
	143	1	0.200	0 ==> 1
	260	1	0.250	1 ==> 0
node_87 --> node_88	38	1	0.250	0 ==> 1

63 2 0.125 0 ==> 2
164 1 0.222 2 ==> 1
187 1 0.158 1 --> 2
271 1 0.200 1 --> 0
node_88 --> Speonycteris aurantiadens 23 1 0.133 1 ==> 2
27 1 1.000 0 ==> 1
176 1 0.400 0 ==> 1
186 1 0.158 3 ==> 4
187 1 0.158 2 ==> 3
192 1 0.214 3 ==> 1
202 1 0.182 1 ==> 2
203 1 0.250 1 --> 0
220 1 0.111 1 ==> 0
221 1 0.143 1 ==> 0
237 1 0.333 1 ==> 0
252 1 0.182 0 ==> 1
253 1 0.278 1 --> 0
264 1 0.200 0 ==> 1
node_88 --> Micronycteris hirsuta 26 1 0.250 0 ==> 1
31 1 0.286 1 ==> 2
34 1 0.158 0 --> 1
53 1 0.125 0 --> 1
55 1 0.167 0 --> 1
57 1 0.286 0 --> 1
60 2 0.158 0 --> 2
61 1 0.154 1 ==> 0
64 1 0.667 1 ==> 2
84 1 0.200 2 --> 0
87 1 0.250 0 --> 1
93 1 0.200 0 --> 1
98 1 0.286 4 --> 3
103 1 0.200 1 --> 2
106 2 0.107 1 --> 3
112 1 0.125 1 --> 2
135 1 0.200 0 --> 1
146 1 0.250 1 --> 0
153 1 0.222 1 --> 0
161 1 0.167 0 --> 1
162 1 0.364 2 ==> 1
165 1 0.125 1 --> 2
167 1 0.154 2 --> 1
172 1 0.167 0 ==> 1
207 1 0.100 0 --> 1
210 1 0.200 2 --> 1
221 1 0.143 1 ==> 2
253 1 0.278 1 --> 2
263 1 0.222 1 ==> 0
node_87 --> Mimon bennettii 1 1 0.100 1 ==> 0
16 1 0.222 0 --> 1
18 1 0.167 0 ==> 1
24 1 0.100 1 --> 0

	47	1	0.200	1 ==> 0
	49	1	0.286	1 ==> 0
	54	1	0.200	1 ==> 0
	105	1	0.133	1 ==> 0
	106	1	0.107	1 ==> 0
	111	1	0.111	1 ==> 0
	117	1	0.167	1 ==> 0
	120	1	0.143	1 ==> 2
	128	1	0.143	1 ==> 0
	148	2	0.143	2 ==> 0
	150	1	0.200	0 ==> 1
	165	1	0.125	1 --> 2
	167	2	0.154	2 ==> 0
	174	1	0.167	0 ==> 1
	196	1	0.200	1 ==> 0
	203	1	0.250	1 --> 0
	210	1	0.200	2 --> 1
	223	1	0.222	1 ==> 2
	226	1	0.111	0 ==> 1
	232	1	0.200	1 ==> 0
	234	1	0.200	0 ==> 1
	269	1	0.222	0 ==> 1
	278	1	0.091	1 ==> 0
node_86 --> node_71	19	1	0.200	0 --> 1
	23	1	0.133	1 --> 0
	24	1	0.100	1 --> 0
	37	1	0.200	1 ==> 0
	53	1	0.125	0 ==> 1
	56	1	0.250	0 ==> 1
	59	1	0.125	1 --> 0
	63	1	0.125	0 ==> 1
	93	1	0.200	0 --> 1
	98	1	0.286	4 ==> 2
	105	1	0.133	1 ==> 2
	106	2	0.107	1 ==> 3
	118	1	0.375	4 ==> 2
	132	1	0.250	0 ==> 1
	146	1	0.250	1 ==> 0
	161	1	0.167	0 ==> 1
	184	1	0.143	1 --> 0
	186	2	0.158	3 ==> 1
	207	1	0.100	0 --> 1
	208	1	0.250	0 ==> 1
	215	1	0.143	0 ==> 1
	217	1	0.286	2 ==> 1
	220	1	0.111	1 ==> 2
	223	1	0.222	1 ==> 2
	235	1	0.250	1 ==> 2
	237	1	0.333	1 ==> 2
	271	1	0.200	1 --> 0
node_71 --> Macrotus californicus	20	1	0.333	0 ==> 1

61 1 0.154 1 ==> 0
69 1 0.154 1 --> 0
112 1 0.125 1 ==> 0
148 1 0.143 2 ==> 1
170 1 0.333 0 ==> 1
187 1 0.158 1 --> 2
192 1 0.214 3 ==> 2
221 1 0.143 1 ==> 2
226 1 0.111 0 ==> 1
236 1 0.143 0 --> 2
250 1 0.222 1 ==> 0
253 1 0.278 1 ==> 0
node_71 --> Macrotus waterhousii 26 1 0.250 0 ==> 1
34 1 0.158 0 ==> 1
38 1 0.250 0 ==> 1
58 1 0.167 2 ==> 1
78 1 0.200 1 --> 0
87 1 0.250 0 ==> 1
123 1 0.167 1 ==> 2
131 1 0.444 2 ==> 3
182 1 0.200 0 ==> 1
185 1 0.250 0 ==> 1
187 1 0.158 1 ==> 0
195 1 0.182 0 ==> 1
206 1 0.286 2 ==> 1
236 1 0.143 0 --> 1
244 1 0.333 0 ==> 1
249 1 0.111 0 ==> 1
250 1 0.222 1 ==> 2
254 2 0.182 2 ==> 0
270 1 0.333 0 ==> 1
node_86 --> node_85 23 1 0.133 1 --> 0
59 1 0.125 1 --> 0
167 1 0.154 2 --> 1
185 2 0.250 0 ==> 2
node_85 --> node_73 5 1 0.273 1 --> 4
9 1 0.250 0 --> 1
11 1 0.250 1 --> 0
12 5 0.455 0 ==> 5
20 1 0.333 0 ==> 1
21 1 0.125 0 ==> 1
24 1 0.100 1 --> 0
25 1 0.200 2 ==> 1
29 1 0.214 2 ==> 3
31 1 0.286 1 --> 0
38 1 0.250 0 ==> 1
39 1 0.333 0 ==> 1
46 1 0.250 0 ==> 1
61 1 0.154 1 --> 2
64 1 0.667 1 ==> 0
69 1 0.154 1 ==> 2

	70	1	0.400	2 ==> 1
	75	1	0.333	0 ==> 1
	84	1	0.200	2 --> 0
	93	1	0.200	0 --> 1
	98	1	0.286	4 ==> 0
	101	1	0.500	0 ==> 1
	110	3	0.143	1 ==> 4
	114	1	0.250	0 ==> 1
	118	1	0.375	4 ==> 0
	127	3	0.138	1 ==> 4
	130	1	0.333	0 ==> 1
	159	1	0.250	0 ==> 1
	160	1	0.111	0 ==> 1
	162	1	0.364	2 ==> 4
	164	2	0.222	2 ==> 0
	165	1	0.125	1 ==> 0
	167	1	0.154	1 --> 0
	169	1	0.200	0 ==> 1
	175	1	0.143	1 --> 2
	178	1	0.250	0 ==> 1
	184	1	0.143	1 --> 0
	187	1	0.158	1 ==> 0
	189	1	1.000	1 ==> 2
	194	2	0.143	2 --> 0
	195	1	0.182	0 --> 2
	207	1	0.100	0 --> 1
	217	2	0.286	2 ==> 0
	221	1	0.143	1 ==> 0
	223	1	0.222	1 ==> 0
	227	2	0.286	0 ==> 2
	233	1	0.286	1 ==> 2
	236	1	0.143	0 ==> 1
	239	1	1.000	0 ==> 1
	243	2	0.333	0 ==> 2
	245	2	0.222	0 --> 2
	247	1	0.200	1 --> 0
	251	1	0.333	0 ==> 1
	252	2	0.182	0 ==> 2
	253	1	0.278	1 ==> 5
	265	1	0.500	0 ==> 1
node_73 --> node_72	15	1	0.250	0 ==> 1
	105	1	0.133	1 --> 0
	149	1	0.333	0 ==> 1
	153	1	0.222	1 ==> 0
	202	1	0.182	1 ==> 0
	204	1	0.500	1 ==> 0
	257	1	1.000	0 ==> 1
node_72 --> Desmodus rotundus	58	1	0.167	2 ==> 3
	63	1	0.125	0 ==> 1
	67	2	0.143	2 ==> 0
	106	1	0.107	1 ==> 0

	113	1	1.000	0 ==> 1
	146	1	0.250	1 --> 0
	154	1	0.143	0 ==> 1
	158	2	0.200	0 ==> 2
node_72 --> Diaemus youngi	25	1	0.200	1 --> 0
	58	1	0.167	2 --> 1
	70	1	0.400	1 ==> 0
	106	1	0.107	1 --> 2
	145	1	0.667	0 ==> 3
	151	1	0.400	0 ==> 1
	186	1	0.158	3 --> {12}
	210	1	0.200	2 ==> 3
	220	1	0.111	1 --> 0
	222	1	0.500	0 ==> 1
	251	1	0.333	1 --> 2
node_73 --> Diphylla ecaudata	14	1	0.667	0 --> 2
	17	1	0.200	0 --> 1
	25	1	0.200	1 --> 0
	58	1	0.167	2 --> 1
	76	1	0.500	0 ==> 1
	146	1	0.250	1 ==> 2
	152	3	0.333	0 ==> 3
	153	1	0.222	1 ==> 2
	186	2	0.158	3 ==> 1
	220	1	0.111	1 --> 0
	235	1	0.250	1 --> 0
	237	1	0.333	1 --> 0
	251	1	0.333	1 --> 2
	255	1	0.200	1 --> 0
	259	1	0.125	0 --> 1
	260	1	0.250	1 --> 2
	262	3	0.375	0 --> 3
	263	1	0.222	1 --> 2
	264	2	0.200	0 --> 2
node_85 --> node_84	16	1	0.222	0 --> 1
	216	1	1.000	1 --> 0
node_84 --> node_78	19	1	0.200	0 --> 1
	29	1	0.214	2 ==> 1
	31	1	0.286	1 --> 0
	47	1	0.200	1 ==> 2
	50	1	0.400	2 --> 1
	83	1	0.200	0 ==> 1
	106	2	0.107	1 ==> 3
	148	1	0.143	2 --> 1
	175	1	0.143	1 --> 2
	186	1	0.158	3 ==> 4
	187	2	0.158	1 ==> 3
	188	1	0.250	0 ==> 1
	221	1	0.143	1 ==> 2
	260	1	0.250	1 ==> 0
	263	1	0.222	1 ==> 0

node_78 --> node_77 220 1 0.111 1 ==> 2
node_77 --> node_76 54 1 0.200 1 --> 0
84 1 0.200 2 --> 0
143 1 0.200 0 --> 1
185 1 0.250 2 --> 1
271 1 0.200 1 --> 0
node_76 --> Lophostoma evotis 5 1 0.273 1 ==> 0
14 1 0.667 0 ==> 1
25 1 0.200 2 ==> 3
53 1 0.125 0 --> 1
60 2 0.158 0 --> 2
78 1 0.200 1 --> 0
82 1 0.500 0 ==> 1
84 1 0.200 0 ==> 3
97 1 0.125 0 --> 1
100 1 0.333 0 ==> 1
105 1 0.133 1 --> 2
107 1 0.333 1 ==> 0
108 1 0.333 0 ==> 2
111 1 0.111 1 ==> 0
112 1 0.125 1 --> 0
123 1 0.167 1 ==> 2
124 1 0.333 0 ==> 2
134 1 0.200 1 --> 0
146 1 0.250 1 ==> 0
148 1 0.143 1 ==> 2
150 1 0.200 0 ==> 1
161 1 0.167 0 ==> 1
177 2 0.500 0 ==> 2
194 1 0.143 2 --> 1
195 1 0.182 0 ==> 1
214 3 0.333 3 ==> 0
233 1 0.286 1 ==> 0
235 1 0.250 1 ==> 2
259 1 0.125 0 ==> {12}
node_76 --> Phyllostomus hastatus hastatus 2 1 0.167 0 --> 1
33 1 0.500 1 ==> 0
34 1 0.158 0 ==> 1
47 2 0.200 2 ==> 0
59 1 0.125 0 ==> 1
60 2 0.158 0 --> 2
61 1 0.154 1 ==> 0
66 1 0.286 0 ==> 1
67 1 0.143 2 ==> 1
71 1 0.111 2 --> 1
100 1 0.333 0 ==> 2
106 3 0.107 3 ==> 0
112 1 0.125 1 ==> 2
120 1 0.143 1 ==> 0
129 1 0.154 1 --> 2
160 1 0.111 0 ==> 1

167 1 0.154 1 ==> 2
174 1 0.167 0 ==> 1
190 1 0.167 0 ==> 1
194 2 0.143 2 ==> 0
210 1 0.200 2 ==> 1
220 1 0.111 2 ==> 1
221 1 0.143 2 ==> 1
253 1 0.278 1 ==> 0
277 1 0.091 1 ==> 0
node_76 --> node_75 234 1 0.200 0 ==> 1
249 1 0.111 0 ==> 1
node_75 --> Phylloderma stenops stenops 1 1 0.100 1 --> 0
2 1 0.167 0 --> 1
5 1 0.273 1 --> 2
18 1 0.167 0 --> 1
20 1 0.333 0 --> 1
24 1 0.100 1 --> 0
29 2 0.214 1 --> 3
51 1 0.167 0 --> 1
53 1 0.125 0 --> 1
58 1 0.167 2 --> 1
97 1 0.125 0 --> 1
105 1 0.133 1 --> 2
112 1 0.125 1 --> 0
120 1 0.143 1 --> 2
123 1 0.167 1 ==> 2
129 1 0.154 1 ==> 0
134 1 0.200 1 --> 0
145 1 0.667 0 --> 2
151 1 0.400 0 --> 1
153 1 0.222 1 --> 0
154 1 0.143 0 --> 1
162 1 0.364 2 --> 1
165 1 0.125 1 --> 0
179 1 0.333 1 --> 2
185 1 0.250 1 --> 0
226 1 0.111 0 ==> 1
278 1 0.091 1 --> 0
node_75 --> node_74 233 1 0.286 1 ==> 0
node_74 --> Notonycteris magdalenensis 78 1 0.200 1 --> 0
115 1 0.333 1 --> 0
118 1 0.375 4 --> 1
119 1 0.333 0 --> 1
122 1 0.250 3 --> 4
123 1 0.167 1 --> 0
127 1 0.138 1 --> 0
129 1 0.154 1 --> 2
243 1 0.333 0 ==> 1
263 1 0.222 0 ==> 1
node_77 --> Tonatia saurophila saurophila 190 1 0.167 0 ==> 1
195 1 0.182 0 ==> 1

	202	1	0.182	1 ==> 0
node_78 --> Trachops cirrhosus cirrhosus	5	1	0.273	1 --> 2
	14	1	0.667	0 ==> 1
	16	1	0.222	1 ==> 2
	26	2	0.250	0 ==> 2
	34	1	0.158	0 ==> 1
	58	1	0.167	2 ==> 1
	61	1	0.154	1 ==> 0
	66	1	0.286	0 --> 1
	71	1	0.111	2 --> 1
	78	1	0.200	1 --> 0
	85	1	0.200	1 ==> 0
	86	1	0.400	0 ==> 1
	87	1	0.250	0 ==> 1
	100	1	0.333	0 ==> 3
	103	1	0.200	1 ==> 2
	108	1	0.333	0 ==> 1
	111	1	0.111	1 ==> 0
	112	1	0.125	1 ==> 2
	120	1	0.143	1 ==> 2
	122	1	0.250	3 ==> 4
	124	1	0.333	0 ==> 1
	129	1	0.154	1 --> 2
	140	1	0.222	2 ==> 1
	152	1	0.333	0 ==> 1
	165	1	0.125	1 ==> 2
	177	1	0.500	0 ==> 1
	179	1	0.333	1 --> 2
	182	1	0.200	0 ==> 1
	192	1	0.214	3 ==> 1
	193	1	0.200	1 ==> 0
	203	1	0.250	1 ==> 0
	210	1	0.200	2 ==> 1
	220	1	0.111	1 --> 0
	223	1	0.222	1 ==> 2
	235	1	0.250	1 ==> 2
	236	1	0.143	0 ==> 2
	237	1	0.333	1 ==> 2
	267	1	0.154	1 ==> 0
	269	1	0.222	0 --> 1
	277	1	0.091	1 ==> 0
	278	1	0.091	1 ==> 0
node_84 --> node_83	11	1	0.250	1 --> 0
	61	1	0.154	1 --> 2
	65	1	0.200	1 ==> 0
	66	1	0.286	0 --> 1
	123	1	0.167	1 ==> 0
	127	1	0.138	1 --> 2
	134	1	0.200	1 --> 0
	184	1	0.143	1 --> 0
	194	2	0.143	2 --> 0

	195	1	0.182	0 --> 2
	219	1	0.125	0 ==> 1
	245	2	0.222	0 --> 2
	247	1	0.200	1 --> 0
	252	1	0.182	0 --> 1
	260	1	0.250	1 --> 2
	264	1	0.200	0 --> 1
	269	2	0.222	0 ==> 2
	274	1	0.250	2 ==> 1
node_83 --> node_81	13	1	0.273	3 --> 2
	21	1	0.125	0 ==> 1
	29	1	0.214	2 ==> 3
	34	1	0.158	0 ==> 1
	37	1	0.200	1 ==> 0
	49	1	0.286	1 --> 2
	50	2	0.400	2 ==> 0
	55	1	0.167	0 --> 1
	60	1	0.158	0 --> 1
	63	1	0.125	0 ==> 1
	105	1	0.133	1 ==> 2
	110	1	0.143	1 --> 2
	122	1	0.250	3 --> 2
	131	1	0.444	2 ==> 1
	139	3	0.227	5 ==> 2
	140	1	0.222	2 ==> 1
	159	1	0.250	0 ==> 1
	160	1	0.111	0 ==> 1
	167	1	0.154	1 --> 0
	172	1	0.167	0 ==> 1
	173	1	0.286	0 --> 1
	178	1	0.250	0 ==> 1
	180	1	0.500	0 ==> 1
	182	1	0.200	0 ==> 1
	192	1	0.214	3 ==> 0
	206	1	0.286	2 ==> 1
	253	1	0.278	1 --> 4
	255	1	0.200	1 --> 0
	271	1	0.200	1 --> 0
	275	1	0.333	1 --> 2
	280	1	0.125	2 ==> 1
node_81 --> node_80	106	2	0.107	1 ==> 3
	112	1	0.125	1 --> 0
	129	1	0.154	1 --> 0
	165	1	0.125	1 ==> 0
	203	1	0.250	1 ==> 0
	235	1	0.250	1 ==> 0
	236	1	0.143	0 ==> 1
	259	1	0.125	0 ==> 1
node_80 --> Anoura geoffroyi peruana	1	1	0.100	1 --> 0
	13	1	0.273	2 --> 1
	25	2	0.200	2 --> 0

	40	3	0.231	3 ==> 0
	41	1	1.000	0 --> 1
	42	1	1.000	1 --> 0
	56	1	0.250	0 ==> 1
	63	1	0.125	1 --> 2
	70	1	0.400	2 ==> 1
	82	1	0.500	0 ==> 1
	84	1	0.200	2 ==> 3
	85	1	0.200	1 ==> 0
	87	1	0.250	0 ==> 1
	111	1	0.111	1 ==> 0
	114	1	0.250	0 --> 1
	128	1	0.143	1 --> 0
	131	1	0.444	1 ==> 0
	138	1	1.000	1 ==> 0
	140	1	0.222	1 --> 0
	144	1	1.000	0 ==> 1
	150	1	0.200	0 ==> 1
	164	1	0.222	2 --> 1
	170	1	0.333	0 --> 1
	173	1	0.286	1 ==> 2
	176	1	0.400	0 ==> 1
	186	1	0.158	3 ==> 2
	193	1	0.200	1 ==> 0
	202	1	0.182	1 ==> 2
	206	1	0.286	1 ==> 0
	210	2	0.200	2 ==> 0
	219	1	0.125	1 ==> 0
	244	1	0.333	0 ==> 1
	249	1	0.111	0 --> 1
	253	1	0.278	4 ==> 3
	263	1	0.222	1 ==> 0
	267	1	0.154	1 ==> 0
	278	1	0.091	1 ==> 0
node_80 --> node_79	12	1	0.455	0 --> 1
	23	1	0.133	0 ==> 1
	26	1	0.250	0 --> 1
	58	1	0.167	2 ==> 1
	145	1	0.667	0 --> 4
	148	1	0.143	2 --> 1
	149	1	0.333	0 --> 1
	151	1	0.400	0 --> 3
	154	1	0.143	0 --> 1
	158	1	0.200	0 --> 1
	220	1	0.111	1 --> 0
	243	1	0.333	0 ==> 1
node_79 --> Phyllonycteris aphylla	1	1	0.100	1 --> 0
	5	1	0.273	1 ==> 2
	29	1	0.214	3 ==> 2
	31	1	0.286	1 ==> 0
	34	1	0.158	1 ==> 0

37	1	0.200	0 ==> 1
47	1	0.200	1 ==> 2
53	1	0.125	0 ==> 1
54	1	0.200	1 ==> 0
58	1	0.167	1 ==> 0
63	1	0.125	1 ==> 0
65	1	0.200	0 ==> 1
66	1	0.286	1 ==> 0
68	1	0.500	0 ==> 1
75	1	0.333	0 ==> 1
76	1	0.500	0 ==> 1
81	2	0.500	1 ==> 3
93	1	0.200	0 ==> 1
98	1	0.286	4 --> 1
102	1	0.200	2 ==> 1
110	1	0.143	2 ==> 1
114	1	0.250	0 --> 1
120	1	0.143	1 --> 0
127	2	0.138	2 ==> 0
133	1	0.500	0 --> 1
139	2	0.227	2 --> 0
142	1	0.500	0 --> 1
153	1	0.222	1 ==> 2
172	1	0.167	1 ==> 0
174	1	0.167	0 ==> 1
192	1	0.214	0 ==> 3
202	1	0.182	1 ==> 0
204	1	0.500	1 ==> 2
207	1	0.100	0 ==> 1
211	1	0.500	0 ==> 1
217	1	0.286	2 ==> 1
221	1	0.143	1 ==> 0
223	1	0.222	1 ==> 0
233	1	0.286	1 ==> 2
234	1	0.200	0 ==> 1
237	1	0.333	1 ==> 0
238	1	0.500	0 ==> 1
243	1	0.333	1 ==> 2
252	1	0.182	1 ==> 2
253	1	0.278	4 --> 2
259	1	0.125	1 ==> 2
262	2	0.375	0 ==> 2
264	1	0.200	1 ==> 2
272	1	0.500	0 --> 1
274	1	0.250	1 --> 0
277	1	0.091	1 --> 0
node_79 --> Leptonycteris yerbabuenae 13	1	0.273	2 --> 1
19	1	0.200	0 ==> 1
23	1	0.133	1 ==> 2
26	1	0.250	1 ==> 2
31	1	0.286	1 ==> 2

	34	1	0.158	1 ==> 2
	36	1	0.333	1 --> 0
	63	1	0.125	1 --> 2
	84	1	0.200	2 --> 0
	97	1	0.125	0 --> 1
	98	1	0.286	4 --> 3
	110	2	0.143	2 ==> 4
	115	1	0.333	1 --> 0
	118	1	0.375	4 ==> 2
	121	1	0.500	0 ==> 1
	122	2	0.250	2 ==> 0
	127	2	0.138	2 ==> 4
	130	1	0.333	0 ==> 1
	163	1	0.200	0 ==> 1
	164	1	0.222	2 --> 1
	166	1	1.000	0 ==> 1
	170	1	0.333	0 --> 1
	204	1	0.500	1 ==> 0
	206	1	0.286	1 ==> 2
	222	1	0.500	0 ==> 1
	250	1	0.222	1 ==> 2
	265	1	0.500	0 ==> 1
node_81 --> Lonchophylla thomasi	17	1	0.200	0 ==> 1
	24	1	0.100	1 --> 0
	25	2	0.200	2 --> 0
	31	1	0.286	1 ==> 2
	34	1	0.158	1 ==> 2
	53	1	0.125	0 ==> 1
	54	1	0.200	1 ==> 0
	60	1	0.158	1 ==> 2
	71	1	0.111	2 --> 1
	72	1	1.000	0 ==> 1
	110	2	0.143	2 ==> 4
	117	1	0.167	1 --> 0
	120	1	0.143	1 --> 0
	127	2	0.138	2 ==> 4
	139	1	0.227	2 --> 1
	140	1	0.222	1 --> 0
	152	1	0.333	0 --> 1
	183	1	1.000	1 ==> 0
	187	1	0.158	1 --> 2
	249	1	0.111	0 --> 1
	250	1	0.222	1 ==> 2
node_83 --> node_82	51	1	0.167	0 ==> 1
	59	1	0.125	0 ==> 1
	69	1	0.154	1 ==> 0
	71	1	0.111	2 --> 1
	81	2	0.500	1 ==> 3
	84	1	0.200	2 --> 0
	86	1	0.400	0 ==> 1
	93	1	0.200	0 --> 1

127 1 0.138 2 --> 3
217 1 0.286 2 ==> 1
220 1 0.111 1 --> 0
227 1 0.286 0 ==> 1
250 1 0.222 1 ==> 0
node_82 --> Carollia perspicillata 2 1 0.167 0 ==> 1
5 1 0.273 1 --> 0
17 1 0.200 0 ==> 1
31 1 0.286 1 --> 0
38 1 0.250 0 ==> 1
49 1 0.286 1 --> 2
55 1 0.167 0 --> 1
56 1 0.250 0 ==> 1
57 2 0.286 0 ==> 2
86 1 0.400 1 ==> 2
98 1 0.286 4 --> 1
102 1 0.200 2 --> 1
103 1 0.200 1 --> 0
105 1 0.133 1 ==> 0
110 3 0.143 1 ==> 4
118 1 0.375 4 --> 1
119 1 0.333 0 --> 1
127 1 0.138 3 ==> 4
132 1 0.250 0 --> 1
142 1 0.500 0 ==> 1
146 1 0.250 1 --> 0
153 1 0.222 1 ==> 0
158 1 0.200 0 ==> 1
162 1 0.364 2 --> 1
169 1 0.200 0 ==> 1
176 1 0.400 0 --> 1
186 1 0.158 3 --> 2
202 1 0.182 1 ==> 0
207 1 0.100 0 ==> 1
210 1 0.200 2 ==> 1
214 1 0.333 3 --> 2
240 1 0.250 2 --> 1
243 1 0.333 0 ==> 1
251 1 0.333 0 ==> 1
253 1 0.278 1 --> 2
254 1 0.182 2 ==> 1
255 1 0.200 1 --> 0
262 2 0.375 0 ==> 2
276 1 0.500 0 ==> 1
node_82 --> Artibeus jamaicensis 1 1 0.100 1 ==> 0
5 1 0.273 1 --> 2
12 1 0.455 0 ==> 1
13 1 0.273 3 --> 2
16 1 0.222 1 ==> 0
23 1 0.133 0 ==> 1
24 1 0.100 1 --> 0

32	1	1.000	1 ==> 2
47	1	0.200	1 ==> 2
58	1	0.167	2 ==> 3
60	2	0.158	0 ==> 2
64	1	0.667	1 ==> 2
66	1	0.286	1 ==> 2
67	1	0.143	2 ==> 1
75	1	0.333	0 ==> 1
78	1	0.200	1 ==> 2
98	1	0.286	4 --> 0
101	1	0.500	0 ==> 1
104	1	1.000	0 ==> 1
106	1	0.107	1 ==> 0
107	1	0.333	1 ==> 0
110	1	0.143	1 ==> 0
112	1	0.125	1 --> 2
114	1	0.250	0 ==> 1
118	1	0.375	4 --> 0
121	1	0.500	0 ==> 1
122	3	0.250	3 ==> 0
126	1	0.500	1 ==> 0
128	1	0.143	1 --> 0
131	2	0.444	2 ==> 4
133	1	0.500	0 ==> 1
145	1	0.667	0 ==> 2
148	1	0.143	2 --> 1
151	1	0.400	0 ==> 1
162	1	0.364	2 --> 3
165	1	0.125	1 ==> 0
167	1	0.154	1 --> 0
174	1	0.167	0 ==> 1
192	1	0.214	3 ==> 1
193	1	0.200	1 ==> 0
195	1	0.182	2 ==> 1
196	1	0.200	1 --> 0
221	1	0.143	1 ==> 0
223	1	0.222	1 ==> 0
233	1	0.286	1 ==> 2
235	1	0.250	1 ==> 0
237	1	0.333	1 ==> 0
238	1	0.500	0 ==> 1
244	1	0.333	0 ==> 1
252	1	0.182	1 ==> 2
253	1	0.278	1 --> 0
259	1	0.125	0 ==> 1
263	1	0.222	1 ==> 2
264	1	0.200	1 ==> 2
266	1	0.500	0 ==> 1
267	1	0.154	1 ==> 2
268	1	0.500	0 ==> 1
271	1	0.200	1 ==> 2

272 1 0.500 0 ==> 1
274 1 0.250 1 ==> 0
277 1 0.091 1 --> 0
node_70 --> node_49 5 1 0.273 0 --> 2
84 1 0.200 2 --> 0
116 1 0.167 0 ==> 1
135 1 0.200 0 ==> 1
160 1 0.111 0 ==> 1
250 1 0.222 1 ==> 0
275 1 0.333 1 --> 2
node_49 --> Mormoops blainvillei 11 1 0.250 1 ==> 0
16 1 0.222 0 --> 1
21 1 0.125 0 ==> 1
23 1 0.133 1 ==> 2
34 1 0.158 0 ==> 1
47 1 0.200 1 ==> 0
55 1 0.167 0 ==> 1
58 1 0.167 2 ==> 3
67 1 0.143 1 --> 2
69 1 0.154 1 --> 0
70 1 0.400 2 ==> 1
97 1 0.125 0 --> 1
100 1 0.333 0 ==> 3
112 1 0.125 1 ==> 0
120 1 0.143 1 --> 2
122 1 0.250 3 --> 2
124 1 0.333 0 ==> 1
127 1 0.138 1 --> 0
129 1 0.154 1 ==> 0
165 1 0.125 1 --> 2
182 1 0.200 0 ==> 1
184 1 0.143 1 --> 0
190 1 0.167 0 --> 1
195 1 0.182 0 --> 1
208 1 0.250 0 --> 1
210 1 0.200 2 --> 1
232 1 0.200 1 ==> 0
245 1 0.222 0 --> 1
247 1 0.200 1 ==> 0
253 1 0.278 1 ==> 2
267 1 0.154 1 --> 0
269 1 0.222 0 ==> 1
278 1 0.091 1 --> 0
279 1 0.500 1 ==> 0
280 1 0.125 2 ==> 1
node_49 --> node_48 18 1 0.167 0 --> 1
26 1 0.250 0 ==> 1
59 1 0.125 1 --> 0
63 1 0.125 0 ==> 1
96 1 0.333 0 ==> 1
102 1 0.200 2 ==> 1

103	1	0.200	1 ==> 0
106	2	0.107	1 ==> 3
111	1	0.111	0 --> 1
112	1	0.125	1 ==> 2
118	1	0.375	4 ==> 2
129	1	0.154	1 ==> 2
148	2	0.143	2 ==> 0
153	1	0.222	1 ==> 0
158	1	0.200	1 --> 0
173	1	0.286	1 --> 0
175	1	0.143	1 ==> 2
179	1	0.333	1 ==> 2
186	1	0.158	3 ==> 4
187	2	0.158	1 ==> 3
188	1	0.250	0 ==> 1
194	1	0.143	2 ==> 1
217	1	0.286	2 ==> 1
221	1	0.143	1 ==> 0
226	1	0.111	0 ==> 1

node_48 --> Pteronotus davyi 16 1 0.222 0 --> 1

53	1	0.125	0 ==> 1
67	1	0.143	1 ==> 0
69	1	0.154	1 --> 0
97	1	0.125	0 --> 1
110	1	0.143	1 ==> 0
122	1	0.250	3 --> 2
127	1	0.138	1 --> 0
139	1	0.227	1 ==> 0
165	1	0.125	1 --> 2
190	1	0.167	0 --> 1
210	1	0.200	2 --> 1
220	1	0.111	1 ==> 0
236	1	0.143	0 --> 2
245	1	0.222	0 --> 1
254	1	0.182	2 ==> 1
267	1	0.154	1 --> 0
270	1	0.333	0 ==> 1

node_48 --> Pteronotus parnellii 19 1 0.200 0 ==> 1

23	1	0.133	1 ==> 0
77	1	0.200	0 ==> 1
98	1	0.286	4 ==> 2
132	1	0.250	0 ==> 1
152	1	0.333	1 ==> 0
161	1	0.167	0 ==> 1
165	1	0.125	1 ==> 0
177	1	0.500	0 ==> 1
192	1	0.214	3 ==> 1
194	1	0.143	1 ==> 0
195	1	0.182	0 --> 1
206	1	0.286	2 ==> 1
208	1	0.250	0 --> 1

	215	1	0.143	0 ==> 1
	236	1	0.143	0 --> 1
	259	1	0.125	0 ==> 1
	260	1	0.250	1 ==> 0
	271	1	0.200	1 ==> 0
	274	1	0.250	2 ==> 1
	278	1	0.091	1 --> 0
node_69 --> node_54	18	1	0.167	0 --> 1
	83	1	0.200	0 ==> 1
	97	1	0.125	0 --> 1
	103	1	0.200	1 ==> 0
	129	1	0.154	1 ==> 2
	172	1	0.167	0 ==> 1
	186	1	0.158	3 ==> 2
node_54 --> node_52	29	1	0.214	1 --> 0
	165	1	0.125	1 --> 0
	245	1	0.222	0 ==> 1
	260	1	0.250	0 --> 1
	269	1	0.222	0 ==> 1
node_52 --> node_50	1	1	0.100	0 --> 1
	9	1	0.250	0 --> 1
	13	1	0.273	2 ==> 3
	46	1	0.250	0 ==> 1
	58	1	0.167	2 --> 3
	60	1	0.158	2 --> 1
	67	1	0.143	1 ==> 0
	74	1	0.200	1 --> 0
	78	1	0.200	1 ==> 0
	85	1	0.200	1 ==> 0
	105	1	0.133	1 --> 0
	108	1	0.333	0 ==> 1
	110	1	0.143	2 ==> 1
	112	1	0.125	1 --> 2
	123	1	0.167	1 --> 0
	124	1	0.333	0 ==> 1
	127	1	0.138	2 ==> 1
	139	1	0.227	1 ==> 2
	145	1	0.667	0 ==> 1
	148	1	0.143	2 ==> 1
	150	1	0.200	0 ==> 1
	158	1	0.200	1 ==> 0
	160	1	0.111	0 ==> 1
	161	1	0.167	0 ==> 1
	174	1	0.167	0 ==> 1
	189	1	1.000	1 ==> 0
	192	1	0.214	3 --> 1
	193	1	0.200	1 ==> 0
	195	1	0.182	0 ==> 1
	198	1	0.333	0 ==> 1
	200	1	0.500	0 --> 1
	207	1	0.100	0 --> 1

217	1	0.286	2 ==> 1
221	1	0.143	1 --> 0
222	1	0.500	0 ==> 1
223	1	0.222	1 ==> 2
224	1	1.000	0 --> 1
231	1	0.333	0 ==> 1
250	1	0.222	1 ==> 0
253	1	0.278	1 --> 0
259	1	0.125	0 --> 1
261	1	1.000	0 ==> 1
271	1	0.200	1 ==> 0
277	1	0.091	1 --> 0

node_50 --> Noctilio albiventris 23 1 0.133 1 --> 2

62	1	0.667	0 ==> 1
90	1	0.500	1 ==> 0
107	1	0.333	1 ==> 0
111	1	0.111	0 ==> 1
126	1	0.500	1 ==> 0
128	1	0.143	0 ==> 1
197	1	0.333	0 ==> 1
219	1	0.125	0 ==> 1
236	1	0.143	0 ==> 2
247	1	0.200	1 ==> 0
259	1	0.125	1 ==> 2

node_50 --> Noctilio leporinus 109 1 0.500 0 ==> 1

110	1	0.143	1 ==> 0
122	1	0.250	3 --> 2
125	1	0.500	0 ==> 1

node_52 --> node_51 2 1 0.167 0 ==> 1

13	1	0.273	2 ==> 1
21	1	0.125	0 ==> 1
24	1	0.100	1 --> 0
59	1	0.125	1 ==> 0
61	1	0.154	1 ==> 0
65	1	0.200	1 ==> 0
67	1	0.143	1 --> 2
92	1	0.250	0 ==> 1
96	1	0.333	0 --> 1
110	2	0.143	2 ==> 4
116	1	0.167	0 ==> 1
122	1	0.250	3 --> 2
127	2	0.138	2 ==> 4
155	1	0.200	1 --> 0
158	1	0.200	1 ==> 2
162	1	0.364	2 ==> 0
163	1	0.200	0 --> 1
164	1	0.222	2 ==> 1
175	1	0.143	1 --> 2
202	1	0.182	0 --> 1
205	1	1.000	0 --> 1
215	1	0.143	1 ==> 0

	220	1	0.111	0 --> 1
	226	1	0.111	0 --> 1
	240	1	0.250	2 ==> 1
	242	1	0.333	1 --> 2
	264	1	0.200	0 ==> 1
	274	1	0.250	2 ==> 1
	278	1	0.091	1 --> 0
node_51 --> Furipterus horrens	23	1	0.133	1 --> 2
	34	3	0.158	0 ==> 3
	35	1	0.500	0 --> 1
	55	1	0.167	0 ==> 1
	63	2	0.125	0 ==> 2
	69	1	0.154	1 ==> 2
	77	1	0.200	0 ==> 1
	100	1	0.333	0 --> 1
	106	1	0.107	1 ==> 0
	131	1	0.444	1 ==> 0
	135	1	0.200	0 ==> 1
	143	1	0.200	0 ==> 1
	156	1	0.500	1 --> 2
	172	1	0.167	1 ==> 0
	187	1	0.158	1 --> 2
	192	1	0.214	3 --> 0
	196	1	0.200	0 --> 1
	227	1	0.286	0 ==> 1
	232	1	0.200	1 ==> 0
	236	1	0.143	0 ==> 1
	252	1	0.182	0 ==> 1
	253	1	0.278	1 --> 0
	259	1	0.125	0 --> 1
	267	1	0.154	1 --> 0
	280	1	0.125	2 ==> 1
node_51 --> Amorphochilus schnablii	23	1	0.133	1 ==> 0
	60	1	0.158	2 --> 1
	69	1	0.154	1 ==> 0
	74	1	0.200	1 --> 0
	83	1	0.200	1 ==> 0
	100	1	0.333	0 --> 3
	103	1	0.200	0 ==> 1
	105	1	0.133	1 --> 0
	120	1	0.143	0 --> 1
	123	1	0.167	1 --> 0
	186	1	0.158	2 ==> 1
	192	1	0.214	3 --> 2
	277	1	0.091	1 --> 0
	279	1	0.500	1 ==> 0
node_54 --> node_53	1	1	0.100	0 --> 1
	24	1	0.100	1 --> 0
	25	1	0.200	2 ==> 3
	40	3	0.231	3 ==> 0
	51	1	0.167	0 ==> 1

58	1	0.167	2 --> 3
63	1	0.125	0 ==> 1
91	1	1.000	1 ==> 0
96	1	0.333	0 --> 1
105	1	0.133	1 ==> 2
108	1	0.333	0 ==> 2
120	2	0.143	0 ==> 2
124	1	0.333	0 ==> 2
154	1	0.143	0 ==> 1
155	1	0.200	1 --> 0
156	1	0.500	1 --> 2
162	1	0.364	2 ==> 1
163	1	0.200	0 --> 1
184	1	0.143	1 --> 0
187	1	0.158	1 --> 2
196	1	0.200	0 --> 1
210	1	0.200	2 ==> 1
221	1	0.143	1 ==> 2
226	1	0.111	0 --> 1
232	1	0.200	1 ==> 0
242	1	0.333	1 --> 2
249	1	0.111	0 ==> 1
252	1	0.182	0 ==> 1
267	1	0.154	1 --> 0

node_53 --> Thyroptera discifera 9 1 0.250 0 --> 1

56	1	0.250	0 ==> 1
57	1	0.286	0 ==> 1
67	1	0.143	1 --> {02}
100	1	0.333	0 --> 2
106	2	0.107	1 ==> 3
110	1	0.143	2 ==> 3
116	1	0.167	0 ==> 1
123	1	0.167	1 ==> 2
127	1	0.138	2 ==> 3
178	1	0.250	0 ==> 1
180	1	0.500	0 ==> 1
186	1	0.158	2 ==> 1
190	1	0.167	0 ==> 1
192	1	0.214	3 --> 2
204	1	0.500	1 ==> 2
214	3	0.333	3 ==> 0
220	2	0.111	0 ==> 2
250	1	0.222	1 ==> 2
275	1	0.333	1 --> 0

node_53 --> Thyroptera tricolor 8 1 0.500 0 ==> 1

21	1	0.125	0 ==> 1
33	1	0.500	1 ==> 0
69	1	0.154	1 ==> 0
100	1	0.333	0 --> 3
106	1	0.107	1 ==> 0
117	1	0.167	1 ==> 0

139	1	0.227	1 ==> 0
160	1	0.111	0 ==> 1
165	1	0.125	1 --> 0
202	1	0.182	0 --> 1
207	1	0.100	0 --> 1
233	1	0.286	1 ==> 0
236	1	0.143	0 ==> 2
240	1	0.250	2 ==> 1
254	2	0.182	2 ==> 0
255	1	0.200	1 ==> 0
259	1	0.125	0 --> 1
262	1	0.375	0 ==> 1
280	1	0.125	2 ==> 1

node_68 --> node_58 71 1 0.111 2 ==> 1

74	1	0.200	1 --> 0
84	1	0.200	2 --> 0
92	1	0.250	0 ==> 1
150	1	0.200	0 ==> 1
161	1	0.167	0 ==> 1
169	1	0.200	0 ==> 1
175	1	0.143	1 ==> 2
252	1	0.182	0 ==> 1
271	1	0.200	1 ==> 0
275	1	0.333	1 --> 2
278	1	0.091	1 --> 0

node_58 --> node_56 129 1 0.154 1 --> 0

227	1	0.286	0 ==> 1
253	1	0.278	1 --> 2

node_56 --> node_55 15 1 0.250 0 --> 1

25	1	0.200	2 --> 3
29	1	0.214	1 --> 0
51	1	0.167	0 --> 1
53	1	0.125	0 --> 1
57	1	0.286	0 --> 1
69	1	0.154	1 ==> 0
98	1	0.286	4 ==> 3
103	1	0.200	1 ==> 0
148	2	0.143	2 --> 0
197	1	0.333	0 --> 1
198	1	0.333	0 --> 1
207	1	0.100	0 --> 1
264	1	0.200	0 ==> 1
274	1	0.250	2 ==> 1

node_55 --> Vulcanops worthyae 77 1 0.200 0 ==> 1

94	1	0.250	1 ==> 0
103	1	0.200	0 ==> 1
108	1	0.333	0 ==> 3
109	1	0.500	0 --> 1
110	1	0.143	2 ==> 1
112	2	0.125	0 ==> 2
115	1	0.333	1 ==> 0

124 1 0.333 0 ==> 3
125 1 0.500 0 --> 1
127 1 0.138 2 ==> 1
129 2 0.154 0 ==> 2
134 1 0.200 1 ==> 0
139 1 0.227 1 ==> 0
143 1 0.200 0 ==> 1
213 1 1.000 1 ==> 0
214 1 0.333 3 ==> 2
219 1 0.125 0 ==> 1
221 1 0.143 1 ==> 2
223 1 0.222 1 ==> 0
226 1 0.111 0 ==> 1
242 1 0.333 1 --> 2
245 1 0.222 0 ==> 1
249 1 0.111 0 --> 1
254 1 0.182 2 ==> 1
node_55 --> Mystacina robusta 34 1 0.158 1 ==> 2
56 1 0.250 0 ==> 1
61 1 0.154 1 --> 0
71 1 0.111 1 ==> 2
98 1 0.286 3 ==> 4
194 1 0.143 2 ==> 1
200 1 0.500 0 --> 1
220 1 0.111 0 --> 1
236 1 0.143 0 --> 1
253 1 0.278 2 ==> 1
259 1 0.125 0 --> 1
node_55 --> Mystacina tuberculata 23 1 0.133 1 ==> 0
49 1 0.286 0 ==> 1
71 1 0.111 1 ==> 2
94 1 0.250 1 ==> 0
127 1 0.138 2 ==> 3
165 1 0.125 1 ==> 2
167 1 0.154 2 ==> 1
210 1 0.200 2 ==> 1
220 1 0.111 0 --> 1
235 1 0.250 1 ==> 0
236 1 0.143 0 --> 1
249 1 0.111 0 --> 1
259 1 0.125 0 --> 1
node_55 --> Mystacina miocenalis 74 1 0.200 0 ==> 1
90 1 0.500 1 ==> 0
122 1 0.250 3 ==> 2
127 1 0.138 2 ==> 3
node_56 --> Icarops paradox 61 1 0.154 1 --> 0
63 1 0.125 0 ==> 1
67 1 0.143 1 ==> 0
78 1 0.200 1 ==> 2
116 1 0.167 0 ==> 1
117 1 0.167 0 --> 1

	241	1	0.250	0 ==> 1
	267	1	0.154	1 --> 0
	277	1	0.091	1 ==> 0
	280	1	0.125	2 ==> 1
node_58 --> node_57	219	1	0.125	0 ==> 1
	260	1	0.250	0 --> 1
	267	1	0.154	1 --> 0
node_57 --> Icarops aenae	97	1	0.125	0 --> 1
	110	1	0.143	2 --> 1
	253	1	0.278	1 --> 0
	269	1	0.222	0 ==> 1
	280	1	0.125	2 ==> 1
node_67 --> node_66	16	1	0.222	0 --> 1
	18	1	0.167	0 --> 1
	240	1	0.250	2 ==> 1
node_66 --> Natalus stramineus	5	1	0.273	0 ==> 2
	7	1	1.000	0 --> 1
	21	1	0.125	0 --> 1
	23	1	0.133	1 ==> 0
	25	1	0.200	2 ==> 1
	29	1	0.214	1 --> 0
	34	1	0.158	1 ==> 0
	40	2	0.231	2 ==> 0
	47	1	0.200	1 ==> 0
	56	1	0.250	0 ==> 1
	58	1	0.167	2 --> 3
	60	2	0.158	2 ==> 0
	61	1	0.154	1 --> 0
	62	2	0.667	0 ==> 2
	65	1	0.200	1 ==> 0
	67	1	0.143	1 --> 2
	69	1	0.154	1 ==> 0
	85	1	0.200	1 ==> 0
	92	1	0.250	0 ==> 1
	97	1	0.125	0 --> 1
	98	1	0.286	4 ==> 3
	105	1	0.133	1 ==> 2
	108	1	0.333	0 ==> 2
	110	1	0.143	2 --> 3
	116	1	0.167	0 ==> 1
	122	1	0.250	3 --> 2
	127	2	0.138	2 ==> 4
	131	1	0.444	1 ==> 0
	135	1	0.200	0 ==> 1
	139	1	0.227	1 ==> 0
	155	1	0.200	1 --> 0
	160	1	0.111	0 ==> 1
	162	1	0.364	2 --> 3
	165	1	0.125	1 ==> 0
	184	1	0.143	1 ==> 0
	186	2	0.158	3 ==> 1

192	1	0.214	1 ==> 2
207	1	0.100	0 --> 1
236	1	0.143	0 ==> 2
242	1	0.333	1 --> 2
245	1	0.222	0 ==> 1
252	1	0.182	0 ==> 1
259	2	0.125	0 ==> 2
264	1	0.200	0 ==> 1
267	1	0.154	1 --> 0
273	1	1.000	0 ==> 1
275	1	0.333	1 --> 2
278	1	0.091	1 --> 0
280	1	0.125	2 ==> 1

node_66 --> node_65 2 1 0.167 0 ==> 1

9	1	0.250	0 --> 1
40	1	0.231	2 --> 3
47	1	0.200	1 ==> 2
67	1	0.143	1 ==> 0
71	1	0.111	2 ==> 1
77	1	0.200	0 ==> 1
120	1	0.143	0 --> 1
136	1	0.500	1 ==> 0
141	1	0.500	0 --> 1
154	1	0.143	0 ==> 1
158	1	0.200	1 ==> 0
163	1	0.200	0 ==> 1
164	1	0.222	1 ==> 2
169	1	0.200	0 ==> 1
187	2	0.158	0 --> 2
254	2	0.182	2 ==> 0
271	1	0.200	1 ==> 0

node_65 --> Chalinolobus gouldii 12 2 0.455 1 ==> {34}

13	2	0.273	1 --> 3
16	1	0.222	1 --> 2
24	1	0.100	1 ==> 0
29	1	0.214	1 --> 0
48	2	1.000	0 ==> 2
58	1	0.167	2 --> 3
61	1	0.154	1 --> 0
78	1	0.200	1 --> 0
100	1	0.333	0 ==> 3
110	1	0.143	2 --> 3
120	1	0.143	1 ==> 2
127	1	0.138	2 --> 3
140	1	0.222	0 ==> 1
155	1	0.200	1 --> 0
175	1	0.143	1 ==> 2
186	1	0.158	3 ==> 4
187	1	0.158	2 ==> 3
188	1	0.250	0 ==> 1
202	1	0.182	0 ==> 1

	210	1	0.200	2 ==> 3
	219	1	0.125	0 ==> 1
	227	1	0.286	0 ==> 1
	270	1	0.333	0 ==> 1
	278	1	0.091	1 --> 0
node_65 --> Chaerephon jobensis	15	1	0.250	0 ==> 1
	26	1	0.250	0 ==> 1
	37	1	0.200	1 ==> 0
	51	1	0.167	0 --> 1
	84	1	0.200	2 ==> 0
	103	1	0.200	1 ==> 0
	105	1	0.133	1 ==> 0
	106	2	0.107	0 ==> 2
	108	1	0.333	0 ==> 1
	110	1	0.143	2 ==> 1
	111	1	0.111	0 ==> 1
	112	1	0.125	0 --> 1
	122	1	0.250	3 --> 2
	123	1	0.167	1 ==> 0
	124	1	0.333	0 ==> 1
	127	1	0.138	2 ==> 1
	128	1	0.143	0 ==> 1
	139	1	0.227	1 ==> 2
	146	1	0.250	1 ==> 0
	152	1	0.333	1 ==> 0
	162	1	0.364	2 --> 3
	167	1	0.154	2 --> 1
	176	2	0.400	0 --> 2
	182	1	0.200	0 --> 1
	185	1	0.250	0 ==> 1
	190	1	0.167	0 ==> 1
	192	1	0.214	1 --> 3
	195	1	0.182	0 ==> 1
	197	1	0.333	0 ==> 1
	198	1	0.333	0 ==> 1
	215	1	0.143	1 ==> 0
	221	1	0.143	1 ==> 0
	222	1	0.500	0 ==> 2
	226	1	0.111	0 ==> 1
	229	1	1.000	0 ==> 1
	231	1	0.333	0 ==> 1
	249	1	0.111	0 ==> 1
	253	1	0.278	1 ==> 0
	277	1	0.091	0 --> 1
node_64 --> node_62	21	1	0.125	0 --> 1
	39	1	0.333	0 --> 1
	67	1	0.143	1 --> 2
	77	1	0.200	0 ==> 1
	79	1	1.000	0 ==> 1
	155	1	0.200	1 --> 0
	187	2	0.158	0 --> 2

188 1 0.250 0 --> 1
242 1 0.333 1 --> 2
275 1 0.333 1 --> 2
node_62 --> node_61 175 1 0.143 1 ==> 2
186 1 0.158 3 ==> 4
187 1 0.158 2 ==> 3
206 1 0.286 1 --> 2
209 1 1.000 1 ==> 2
221 1 0.143 1 --> 2
227 1 0.286 0 ==> 1
249 1 0.111 0 ==> 1
252 1 0.182 0 ==> 1
264 1 0.200 0 ==> 1
271 1 0.200 1 ==> 0
node_61 --> node_60 215 1 0.143 0 --> 1
278 1 0.091 1 ==> 0
node_60 --> node_59 1 1 0.100 0 --> 1
2 1 0.167 0 --> 1
8 1 0.500 0 --> 1
13 1 0.273 1 --> 0
18 1 0.167 0 --> 1
40 2 0.231 2 --> 0
44 2 1.000 0 --> 2
47 1 0.200 1 --> 2
60 2 0.158 2 --> 0
61 1 0.154 1 --> 0
65 1 0.200 1 --> 0
69 1 0.154 1 --> 2
86 1 0.400 0 --> 1
98 1 0.286 0 --> 2
110 2 0.143 2 --> 4
118 1 0.375 4 --> 2
119 1 0.333 0 --> 1
127 2 0.138 2 --> 4
132 1 0.250 0 --> 1
134 1 0.200 1 --> 0
139 1 0.227 1 --> 0
146 1 0.250 1 --> 0
148 1 0.143 2 --> 1
159 1 0.250 0 --> 1
160 1 0.111 0 --> 1
162 1 0.364 2 --> 3
173 2 0.286 2 --> 0
194 1 0.143 2 --> 1
228 1 1.000 0 ==> 1
277 1 0.091 0 --> 1
280 1 0.125 2 ==> 1
node_59 --> Myzopoda aurita 92 1 0.250 0 ==> 1
94 1 0.250 1 --> 0
152 1 0.333 1 ==> 0
172 1 0.167 0 ==> 1

259 1 0.125 0 ==> 1
267 1 0.154 1 ==> 0
node_59 --> Myzopoda schliemanni 16 1 0.222 0 --> 1
29 1 0.214 1 --> 0
34 1 0.158 1 ==> 0
43 1 0.600 2 ==> 3
78 1 0.200 1 --> 0
154 1 0.143 0 ==> 1
194 1 0.143 1 ==> 0
231 1 0.333 0 ==> 1
241 1 0.250 0 --> 1
254 1 0.182 2 --> 1
node_60 --> Phasmatonycteris butleri 219 1 0.125 0 --> 1
254 1 0.182 2 --> 1
node_61 --> Phasmatonycteris phiomensis 219 1 0.125 0 --> 1
241 1 0.250 0 --> 1
253 1 0.278 1 ==> 2
node_62 --> Saccopteryx bilineata 3 1 1.000 0 ==> 1
17 1 0.200 0 --> 1
23 1 0.133 1 ==> 2
24 1 0.100 1 ==> 0
34 1 0.158 1 ==> 2
43 2 0.600 2 ==> 0
46 1 0.250 0 ==> 1
58 1 0.167 2 ==> 1
59 1 0.125 1 ==> 0
60 1 0.158 2 ==> 3
63 2 0.125 0 ==> 2
66 1 0.286 0 --> 1
69 1 0.154 1 ==> 0
85 1 0.200 1 ==> 0
94 1 0.250 1 --> 0
95 1 1.000 0 ==> 1
102 1 0.200 1 --> 2
103 1 0.200 1 ==> 0
105 2 0.133 0 ==> 2
106 3 0.107 0 ==> 3
108 1 0.333 0 ==> 2
110 1 0.143 2 ==> 1
111 1 0.111 0 --> 1
112 1 0.125 0 --> 1
116 1 0.167 0 ==> 1
117 1 0.167 0 ==> 1
120 1 0.143 0 --> 1
122 1 0.250 3 ==> 2
123 2 0.167 0 ==> 2
124 1 0.333 0 ==> 1
128 1 0.143 0 --> 1
129 1 0.154 1 --> 2
135 1 0.200 0 --> 1
139 1 0.227 1 --> 2

	153	1	0.222	2 --> 1
	163	1	0.200	0 ==> 1
	164	1	0.222	1 ==> 2
	165	1	0.125	1 ==> 2
	167	1	0.154	2 ==> 1
	169	1	0.200	0 ==> 1
	184	1	0.143	1 ==> 0
	193	1	0.200	1 ==> 0
	208	1	0.250	0 ==> 1
	223	1	0.222	1 ==> 2
	226	1	0.111	0 ==> 1
	232	1	0.200	1 ==> 0
	234	1	0.200	1 --> 0
	236	1	0.143	0 ==> 1
	247	1	0.200	1 ==> 0
	250	1	0.222	1 ==> 0
	255	1	0.200	1 ==> 0
	259	1	0.125	0 ==> 1
	267	1	0.154	1 --> 0
	274	1	0.250	1 --> 2
	277	1	0.091	0 --> 1
node_63 --> Hipposideros commersoni	1	1	0.100	0 --> 1
	15	1	0.250	0 ==> 1
	21	1	0.125	0 --> 1
	23	1	0.133	1 ==> 2
	25	1	0.200	2 ==> 3
	34	1	0.158	1 ==> 2
	35	1	0.500	0 --> 1
	36	1	0.333	0 --> 1
	39	1	0.333	0 --> 1
	40	1	0.231	2 ==> 1
	43	2	0.600	2 ==> 0
	46	1	0.250	0 ==> 1
	58	1	0.167	2 ==> 3
	63	1	0.125	0 ==> 1
	66	1	0.286	0 --> 1
	83	1	0.200	0 --> 1
	86	1	0.400	0 --> 1
	98	1	0.286	0 --> 1
	106	1	0.107	0 --> 1
	131	1	0.444	1 --> 2
	139	4	0.227	1 --> 5
	140	2	0.222	0 --> 2
	141	1	0.500	0 --> 1
	143	1	0.200	0 --> 1
	162	1	0.364	2 --> 3
	174	1	0.167	0 ==> 1
	202	1	0.182	0 --> 1
	207	1	0.100	0 --> 1
	220	2	0.111	0 --> 2
	251	2	0.333	0 --> 2

263	2	0.222	0 --> 2
276	1	0.500	0 --> 1

node_63 --> Pteropus scapulatus 12 1 0.455 1 ==> 0

17	1	0.200	0 --> 1
25	2	0.200	2 ==> 0
29	1	0.214	2 ==> 3
34	1	0.158	1 ==> 0
37	1	0.200	1 ==> 0
49	2	0.286	0 --> 2
56	2	0.250	0 --> 2
57	2	0.286	0 --> 2
58	2	0.167	2 ==> 0
59	1	0.125	1 ==> 0
61	1	0.154	1 ==> 2
67	1	0.143	1 --> 2
68	1	0.500	0 ==> 1
70	1	0.400	2 ==> 1
73	1	1.000	0 ==> 1
130	1	0.333	0 ==> 1
145	1	0.667	0 ==> 4
148	1	0.143	2 ==> 1
149	1	0.333	0 ==> 1
151	1	0.400	0 --> 3
159	1	0.250	0 ==> 1
160	1	0.111	0 ==> 1
165	1	0.125	1 ==> 0
167	2	0.154	2 ==> 0
171	1	1.000	0 ==> 1
178	1	0.250	0 --> 1
185	2	0.250	0 --> 2
194	1	0.143	1 ==> 0
195	1	0.182	0 ==> 2
210	1	0.200	1 ==> 0
211	1	0.500	0 ==> 1
235	1	0.250	1 ==> 0
258	1	1.000	0 ==> 1
266	1	0.500	0 ==> 1
267	1	0.154	1 ==> 2
268	1	0.500	0 ==> 1
269	2	0.222	0 --> 2
